# A transcriptome dataset for gonadectomy-induced changes in rat spinal cord

**DOI:** 10.1038/s41597-022-01917-y

**Published:** 2022-12-29

**Authors:** Shawn Miller, Juan E. Abrahante, Avtar Roopra, Brendan J. Dougherty

**Affiliations:** 1 Divisions of Physical Therapy and Rehabilitation Science, Department of Rehabilitation Medicine, University of Minnesota Medical School, Minneapolis, MN 55455 USA; 2 University of Minnesota Informatics Institute, Minneapolis, MN 55455 USA; 3 Department of Neuroscience, University of Wisconsin, Madison, WI 53705 USA

**Keywords:** Neurology, Neurophysiology

## Abstract

Circulating sex steroid hormones are critical for neural function and development of neuroplasticity in many regions of the central nervous system. In the spinal cord, our knowledge of steroid hormone influence mostly derives from mechanistic studies of pain processing in dorsal spinal cord circuits; less is known regarding hormonal influence of ventral spinal motor function. Gonadectomy (surgical removal of the testes in males and ovaries in females) rapidly and persistently reduces circulating sex steroids in both females and males, providing a means to interrogate the role of hormones on neural function. Here we provide a next-generation RNA sequencing (RNA-seq) data set to evaluate the impact of gonadectomy on the transcriptome of ventral spinal cord tissue of adult female and male rats.

## Abstract (summary)

**Table:** 

Measurement(s)	Ventral spinal cord gene expression
Technology Type(s)	RNA Sequencing
Factor Type(s)	Ventral spinal cord gene expression
Sample Characteristic - Organism	Rattus norvegicus
Sample Characteristic - Location	United States

## Background & Summary

Our understanding of how sex steroid hormones influence central nervous system function across the lifespan has rapidly advanced over the past 30 years. The principle steroid hormones estrogen and testosterone are now recognized to exert pleiotropic effects on virtually all areas of the brain and spinal cord^1^. Primarily synthesized in the gonads and adrenal cortex, steroid hormones are released into the blood circulation, readily cross the blood brain barrier, and influence neuronal signaling through nuclear and non-nuclear receptor mechanisms^1–3^. In the spinal cord, steroid hormones directly modulate neurotransmitter release and synaptic neurotransmission^4^, facilitate myelination^5,6^, provide neuroprotection^7–10^, support axonal regeneration^11^ following spinal cord injury, and mediate the induction of neuroplasticity^2,12–18^. This knowledge comes primarily from studies of pain processing in the dorsal spinal cord, hippocampal learning and memory, and from rodent models of neuroprotection following spinal cord injury. Less understood is the direct role steroid hormones play in ventral spinal cord function and spinal motor output.

Gonadectomy, the surgical removal of the primary sex organs (ovaries in females; testes in males), induces a rapid and sustained reduction in circulating steroid hormones^2,17,19^. This relatively simple procedure is routinely used in basic science studies to ascertain the role of steroid hormones on function, particularly in the CNS. However, gonadectomy-induced reductions in steroid hormones are likely to alter the fundamental properties of the neural system being studied, complicating comparisons with gonadally-intact groups. This study used next-generation RNA sequencing (RNA-seq) to evaluate the impact of gonadectomy on the transcriptome of ventral spinal cord tissue of adult female and male rats. These data provide valuable, sex-specific gene expression profiles for the ventral cervical spinal cords of female and male rats and suggest unique, sexually dimorphic transcriptomic changes resulting from gonadectomy.

## Methods

All experimental procedures were approved by the University of Minnesota Institutional Animal Care and Use Committee and conformed to guidelines detailed in the National Institutes of Health Guide for the Care and Use of Laboratory Animals. Pairs of young-adult (3–4 months) Sprague-Dawley rats (Envigo; Indianapolis, IN, USA) were housed in a mixed-sex rodent room within facilities accredited by the Association for Assessment and Accreditation of Laboratory Animal Care. Rats were acclimatized to the rodent facilities for at least 1 week prior to the start of experimentation and were maintained on a 12 hr light-dark cycle (6am – 6 pm) with food and water available *ad libitum*. A total of 22 rats were used for this study: 10 male rats and 12 virgin female rats.

### Gonadectomy

Half of the male rats (n = 5) and half of the female rats (n = 6) underwent gonadectomy surgeries to reduce levels of circulating sex hormones. This is a common procedure to study the impact of circulating sex hormones on neural function and consists of removing the ovaries bilaterally in female rats (ovariectomy) or removing the bilateral testes (castration) in males. The castrated male rats in this study are referred to as gonadectomized (GDX) and females as ovariectomized (OVX).

At least 2 hours prior to surgery, all rats were provided Buprenorphine SR-LAB (1 mg/kg; s.q. ZooPharm; Fort Collins, CO, USA) for long-lasting pain control. For OVX surgeries, female rats were anesthetized with isoflurane in a closed chamber and then maintained via nose cone (2–3% in 50% O_2_) for the duration of surgery. Adequacy of anesthesia was confirmed by the lack of response to toe-pinch and eye-blink reflexes. Following removal of fur and skin cleansing with chlorhexidine (Prevantics; PDI Inc. Orangeburg, NY, USA), bilateral incisions were made through the dorsolateral skin and muscle layers to expose the ovarian fat pads. The ovaries were exteriorized and separated from the uterine horns using electrocautery to minimize bleeding. Uterine horns were then replaced into the abdominal cavity. Muscle layers were approximated and sutured with 4-0 absorbable suture (Covidien, Mansfield, MA, USA), and the skin incision was closed with wound clips (9 mm autoclips, MikRon Precision Inc. Gardena, CA, USA). Control rats (n = 6) were ovarian-intact and age-matched to OVX rats.

The GDX procedure in males was performed using similar surgical preparations. Male rats were anesthetized with isoflurane in a closed chamber and then maintained via nose cone (2–3% in 50% O_2_) for the duration of surgery. Adequacy of anesthesia was confirmed by the lack of response to toe-pinch and eye-blink reflexes. From a supine position, fur was removed over the bilateral testes and skin cleansed with chlorhexidine. Testes were exteriorized via small, bilateral scrotal incisions. The testes were removed using electrocautery to minimize bleeding and vas deferens were replaced back into the scrotal sac. The skin incisions were closed using veterinary surgical glue (Vetbond, 3 M, St. Paul, MN, USA) with special care taken to avoid the penile or anal openings. Control rats (n = 5) had intact testes and were age-matched to GDX rats. Following OVX and GDX surgeries, rats were recovered in temperature-controlled chambers until mobilizing independently, and then were returned to their home cages.

### Ventral cervical spinal cord tissue isolation

**Fig. 1:**
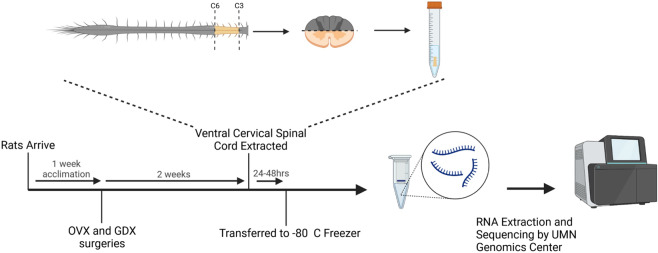
Experimental workflow. Following transcardial perfusion, spinal cord segments from C3-C6 were extracted and the dorsal half of the tissue segments discarded to isolate only the ventral spinal cord. Samples were quickly transferred to ice-cold RNAlater solution and refrigerated at 4 °C for up to 48 hours. The tissue was subsequently removed from RNAlater solution, placed into fresh 1.5mL conical tubes and stored at -80 °C until processed for RNA extraction and sequencing.

Rats were deeply anesthetized with isoflurane in a closed container, transcardially perfused with ice-cold saline (pH 7.4), and the spinal cords immediately extracted from C3-C6 (Fig. 1). The dorsal half of the tissue segments were discarded to isolate only the ventral spinal cord. Samples were quickly transferred to ice-cold RNAlater solution (Invitrogen) and refrigerated at 4 °C for 48 hours. Cords were subsequently removed from RNAlater solution, placed into fresh 1.5 mL conical tubes and stored at −80 °C until processed for RNA extraction (Fig. 1). The ventral cervical spinal cord in this region contains upper motor neurons of UE musculature and the phrenic motor pool, a column of motor neurons innervating the diaphragm muscle via the phrenic nerve. Spinal cord tissue from rats receiving castration or ovariectomy was processed 2 weeks post-surgery (Fig. 1), to align with recent studies indicating that respiratory function and the expression of phrenic neuroplasticity are impacted by gonadectomy in rats^2,16,17^.

### RNA isolation and sequencing

All samples were sent on dry ice to the University of Minnesota Genomic Center for RNA isolation, library preparation and sequencing. RNA was isolated using the RNeasy mini kit (Qiagen Corp., Santa Clarita, CA) according to manufacturer’s instructions. The purity of RNA was checked with Nanodrop (Thermo Fisher Scientific). All RNAs showed a 260:280 ratio of >2.00 (Table 1)

**Table 1:** RNA quantification and quality controls including 260/280 ratios and RNA integrity numbers (RIN#).

Sample	Tissue	RNA Concentration (ng/µL)	260/280 Ratio	RIN #
Intact Female 1	Ventral Spinal Cord - IF1	226	2.09	8.4
Intact Female 2	Ventral Spinal Cord - IF2	102	2.05	8.4
Intact Female 3	Ventral Spinal Cord - IF3	212	2.07	7.8
Intact Female 4	Ventral Spinal Cord - IF4	251	2.07	8.4
Intact Female 5	Ventral Spinal Cord - IF5	141	2.06	7.9
Intact Female 6	Ventral Spinal Cord - IF6	262	2.07	8.6
OVX Female 1	Ventral Spinal Cord - XF1	144	2.08	8.0
OVX Female 2	Ventral Spinal Cord - XF2	177	2.06	8.8
OVX Female 3	Ventral Spinal Cord - XF3	192	2.1	8.1
OVX Female 4	Ventral Spinal Cord - XF4	177	2.07	8.0
OVX Female 5	Ventral Spinal Cord - XF5	230	2.09	8.2
OVX Female 6	Ventral Spinal Cord - XF6	160	2.06	7.9
Intact Male 1	Ventral Spinal Cord - IM1	59	2.08	8.5
Intact Male 2	Ventral Spinal Cord - IM2	176	2.09	8.2
Intact Male 3	Ventral Spinal Cord - IM3	120	2.07	8.4
Intact Male 4	Ventral Spinal Cord - IM5	142	2.05	8.4
Intact Male 5	Ventral Spinal Cord - IM6	225	2.06	8.1
GDX Male 1	Ventral Spinal Cord - XM1	181	2.09	8.2
GDX Male 2	Ventral Spinal Cord - XM2	190	2.04	8.3
GDX Male 3	Ventral Spinal Cord - XM4	295	2.06	8.4
GDX Male 4	Ventral Spinal Cord - XM5	262	2.08	8.0
GDX Male 5	Ventral Spinal Cord - XM6	218	2.07	8.0

**Fig. 2:**
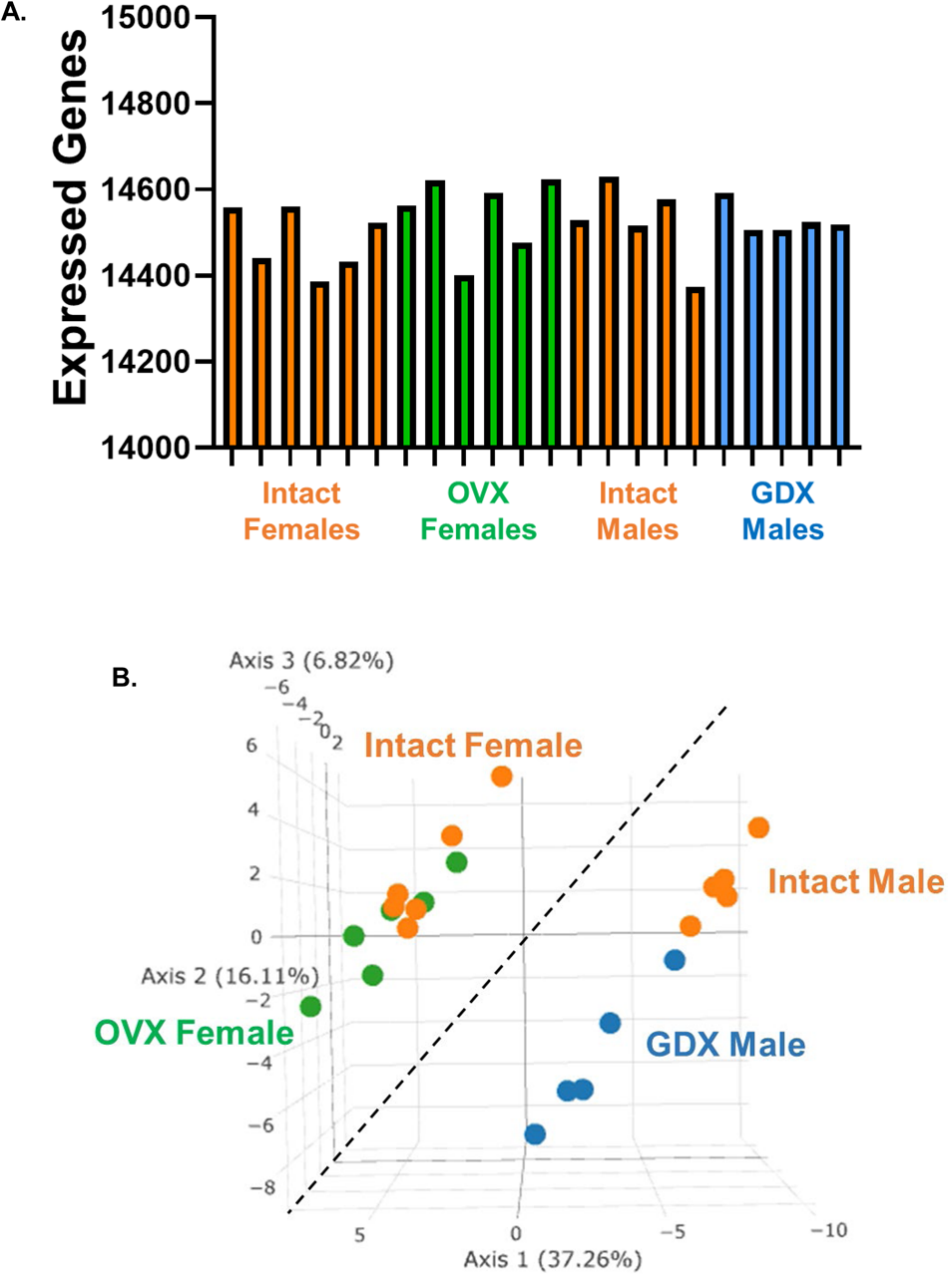
RNA-Seq data analysis. After quality check, reads were mapped to the rat genome (Rnor_6.0.98) using Hisat2 (v2.1.0). The number of genes expressed per sample is shown in (A). For this plot a gene was considered expressed if it had expression equal to or higher than 1 read per million. Principle component analysis (PCA; B) illustrates the clustering of female and male samples. The first three principal components are shown, and the percent of total variation explained by each component is shown in the axis titles. Samples with similar characteristics appear close to each other, and samples with dissimilar characteristics are farther apart.

**Fig. 3:**
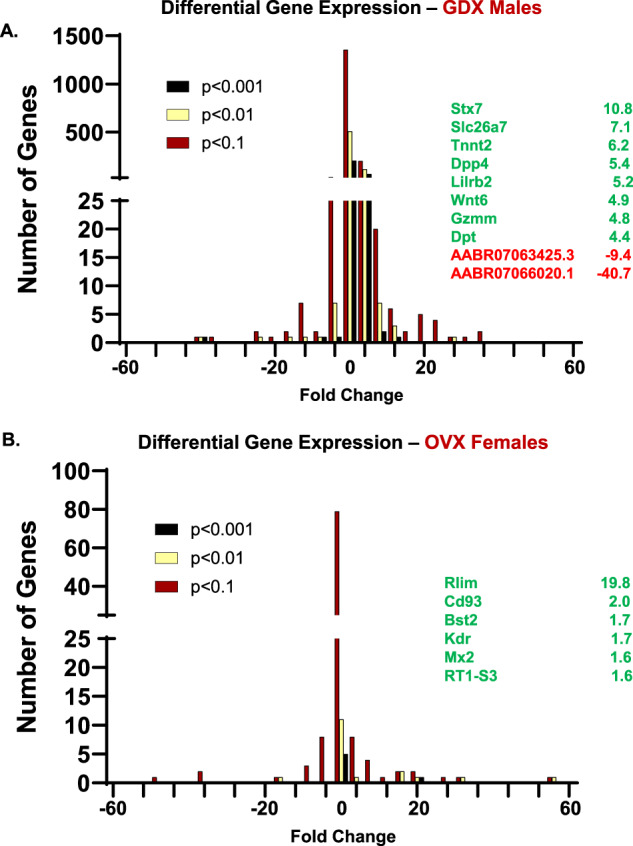
Differential Gene Expression following GDX. Removal of the primary sex organs induced transcriptomic changes in the ventral cervical spinal cord. The frequency distribution of DEGs relative to their fold-change is plotted at three levels of statistical stringency (p < 0.1; p < 0.01; p < 0.0001) in male rats following GDX (A) and female rats following OVX (B). Included are the top 10 most differentially expressed genes at p < 0.001 for males, and the 6 genes upregulated in females at p < 0.001.

RNA isolates were quantified using a fluorimetric RiboGreen assay and RNA integrity was assessed using capillary electrophoresis (Agilent BioAnalyzer 2100) to generate an RNA integrity number (RIN). All samples had RIN values ≥7.8 (avg. RIN 8.2; Table 1) and at least 1 ng total RNA. Sequencing libraries were generated using a SMARTer Stranded Total RNA Seq v2 – Pico Mammalian kit (Takara Bio). Briefly, >1 ng of total RNA were fragmented and then reverse transcribed into cDNA using random primers, with a template switching oligo incorporated during cDNA synthesis to allow for full length cDNA synthesis and retain strand specificity. Illumina sequencing adapters and barcodes were then added to the cDNA by PCR, followed by cleavage of ribosomal cDNA. Uncleaved fragments were then enriched by PCR for 12–16 cycles. Final library size distribution was again validated using capillary electrophoresis and quantified using fluorimetry (PicoGreen). Indexed libraries were normalized and pooled for sequencing. Libraries were then loaded onto a NextSeq. 550 (single read, Illumina) cartridge, where clustering occurred on-board the instrument. After clustering, sequencing was commenced using Illumina’s 2-color SBS chemistry. Following sequencing, Base call (.bcl) files for each cycle of sequencing were generated by Illumina Real Time Analysis software. Primary analysis and de-multiplexing were performed using Illumina bcl2fastq software. The end result of the bcl2fastq workflow was de-multiplexed FASTQ files for subsequent analysis. 151 bp fastq single-end reads (mean read depth ≥40 million per sample) were trimmed using Trimmomatic (version 0.33) enabled with the optional “-q” option; 3 bp sliding-window trimming from 3′ end required minimum Q30. Quality control of raw sequence data for each sample was performed using FastQC (Supplementary Figs. 1–22). Read mapping was performed via Hisat2 (v2.1.0) using the rat genome (Rnor_6.0.98) as reference. Mapping results are provided in Table 2. Average read-pairs per sample was 44.4 M with an average input fragment size of 188 bp (Table 2). Gene quantification was performed using Subread featureCounts for raw read counts (Fig. 2A). Sample variance was identified using principle component analysis (Fig. 2B). Clear separation between male and female samples indicate highly sex-specific expression profiles, and samples tended to cluster based on treatment (i.e. gonadally-intact vs. gonads removed; Fig. 2B). The impact of gonadectomy on gene expression is presented in Fig. 3. Differentially expressed genes (DEGs) were identified using the edgeR (negative binomial) feature in CLCGWB (Qiagen, Redwood City, CA) using raw read counts. The frequency distribution of DEGs relative to their fold-change are plotted at three levels of statistical stringency (p < 0.1; p < 0.01; p < 0.0001; Fig. 3). Removal of the gonads appeared to have a more robust effect on the spinal transcriptome of male rats as a total of 1642 genes were significantly up or down regulated at p < 0.1, while 273 genes remained significantly different at p < 0.001 (Fig. 3A). Of these, 191 genes were significantly upregulated and 82 downregulated. Female gene expression in the ventral cervical spinal cord was minimally affected by removal of the gonads. Only 114 genes were significantly changed following OVX at lower statistical stringency (p < 0.1), with 6 DEGs remaining significantly upregulated at p < 0.001 (Fig. 3B).

**Table 2:** RNA-Seq mapping results and NCBI data accession codes for each sample.

Sample	Tissue	Average input fragment size (bp)	Raw Read-pairs (per million)	% sequence alignment	NCBI SRA Data Accession
Intact Female 1	Ventral Spinal Cord - IF1	162	51.8	98.03	GSM6112668
Intact Female 2	Ventral Spinal Cord - IF2	243	41.7	97.97	GSM6112669
Intact Female 3	Ventral Spinal Cord - IF3	245	47.6	97.75	GSM6112670
Intact Female 4	Ventral Spinal Cord - IF4	162	43.6	97.51	GSM6112671
Intact Female 5	Ventral Spinal Cord - IF5	244	43.6	97.88	GSM6112672
Intact Female 6	Ventral Spinal Cord - IF6	244	38.7	97.77	GSM6112673
OVX Female 1	Ventral Spinal Cord - XF1	162	50.5	97.47	GSM6112674
OVX Female 2	Ventral Spinal Cord - XF2	162	46.6	97.65	GSM6112675
OVX Female 3	Ventral Spinal Cord - XF3	161	41.1	97.75	GSM6112676
OVX Female 4	Ventral Spinal Cord - XF4	162	50.7	97.56	GSM6112677
OVX Female 5	Ventral Spinal Cord - XF5	162	39.6	97.51	GSM6112678
OVX Female 6	Ventral Spinal Cord - XF6	162	37.6	97.59	GSM6112679
Intact Male 1	Ventral Spinal Cord - IM1	243	43.7	97.25	GSM6112658
Intact Male 2	Ventral Spinal Cord - IM2	162	39.8	97.72	GSM6112659
Intact Male 3	Ventral Spinal Cord - IM3	244	39.3	97.87	GSM6112660
Intact Male 4	Ventral Spinal Cord - IM5	162	47.7	97.82	GSM6112661
Intact Male 5	Ventral Spinal Cord - IM6	243	44.3	97.53	GSM6112662
GDX Male 1	Ventral Spinal Cord - XM1	162	44.4	97.65	GSM6112663
GDX Male 2	Ventral Spinal Cord - XM2	162	50.9	97.43	GSM6112664
GDX Male 3	Ventral Spinal Cord - XM4	161	44.0	97.71	GSM6112665
GDX Male 4	Ventral Spinal Cord - XM5	162	42.7	97.93	GSM6112666
GDX Male 5	Ventral Spinal Cord - XM6	162	46.1	97.75	GSM6112667

## Data Records

Tables 1, 2 provide specific information related to each sample used in this study. Complete RNA-seq data were deposited in the NCBI’s Gene Expression Omnibus (GEO) database (GSE202381)^20^.

## Technical Validation

As our samples represent heterogeneous populations of cells in the spinal cord, we eliminated the dorsal spinal tissue in each sample to reduce variability and focus our analysis on the area of primary interest, the ventral cervical spinal cord. The quality of RNA was examined using an Agilent BioAnalyzer 2100 and all samples showed high RNA integrity (Avg. RIN 8.2) appropriate for deep sequencing. RNA-seq data quality was determined using FastQC. Average input fragment size per sample was ~188 bp with a mean read depth of 44.36 million (Table 1). Subsequently, a high percentage of reads were mapped to the reference rat genome (97.69% alignment; Table 1). PCA plots for the 4 groups are provided in Fig. 2.

## Supplementary information

Supplementary Figures

## Data Availability

No custom code was generated or used for analysis of the data presented.
