# Supplementary material for: A transcriptome dataset for gonadectomy-induced changes in rat spinal cord
Source: Sci Data. 2022 Dec 29;9:789. doi: 10.1038/s41597-022-01917-y (PMC9800375; doi:10.1038/s41597-022-01917-y)
Supplement: Supplementary file 1 [file 41597_2022_1917_MOESM1_ESM.docx]

1a

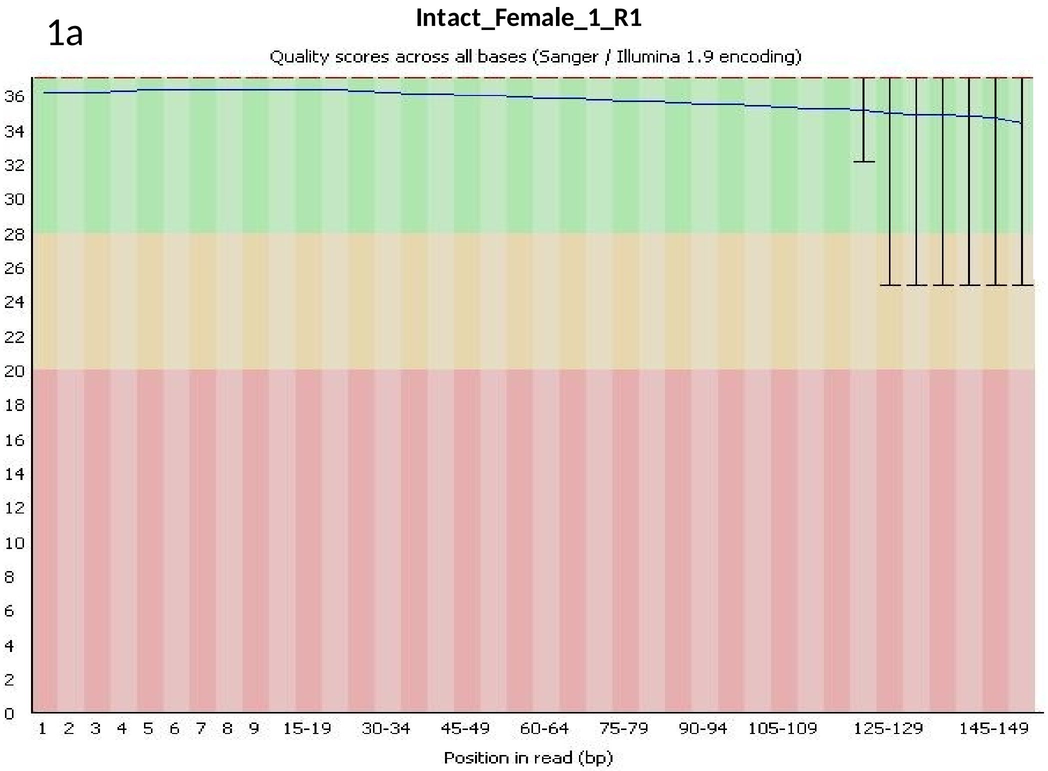

Intact_Female_1

1b

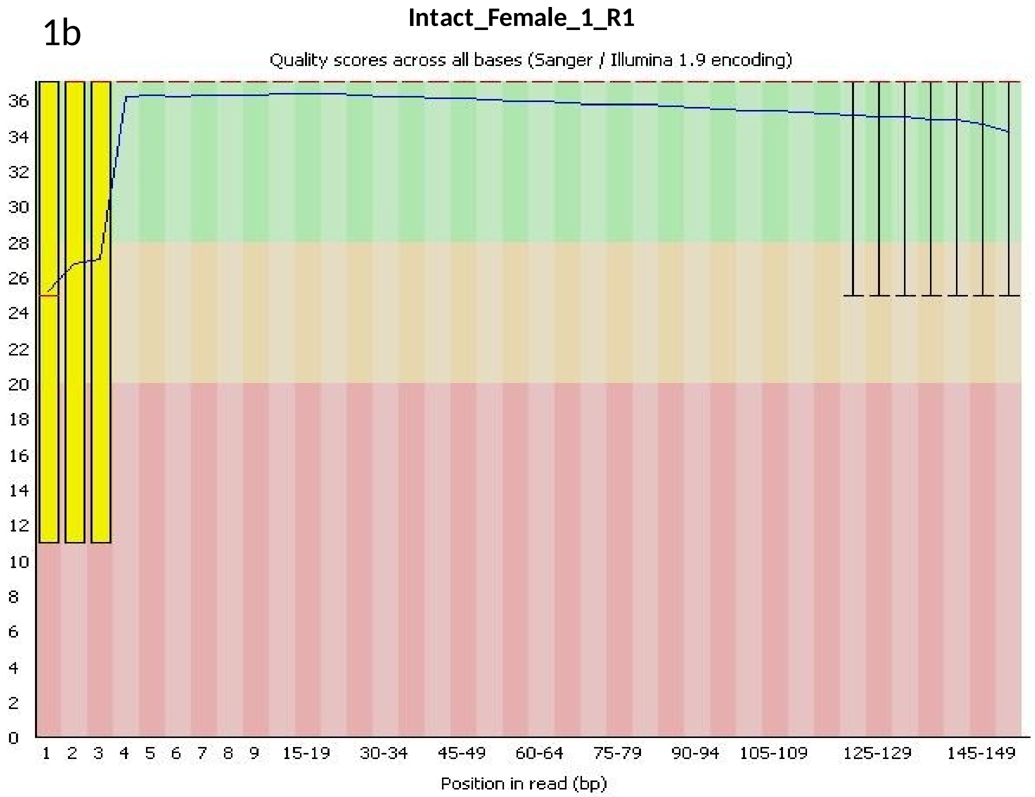

Intact_Female_1_R2

<u>Supplementary Figures 1a and 1b Intact Female #1</u>. FASTQ quality assessment.
Box and whisker plots illustrate the distribution of per base quality for left (R1; 1a) and right (R2; 1b) position reads. The blue line indicates mean value and the yellow box represents interquartile range (25%-75%). Upper and lower whiskers indicate 10% and 90% points respectively. Plots were generated by FASTQC program.

2a

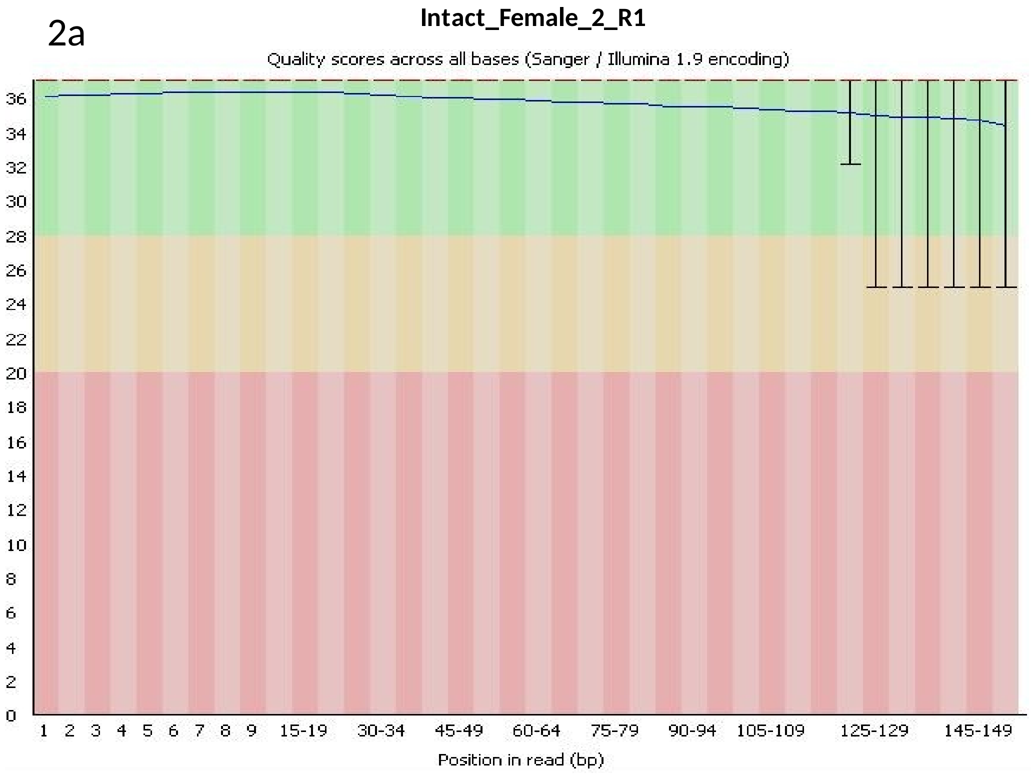

Intact_Female_2

2b

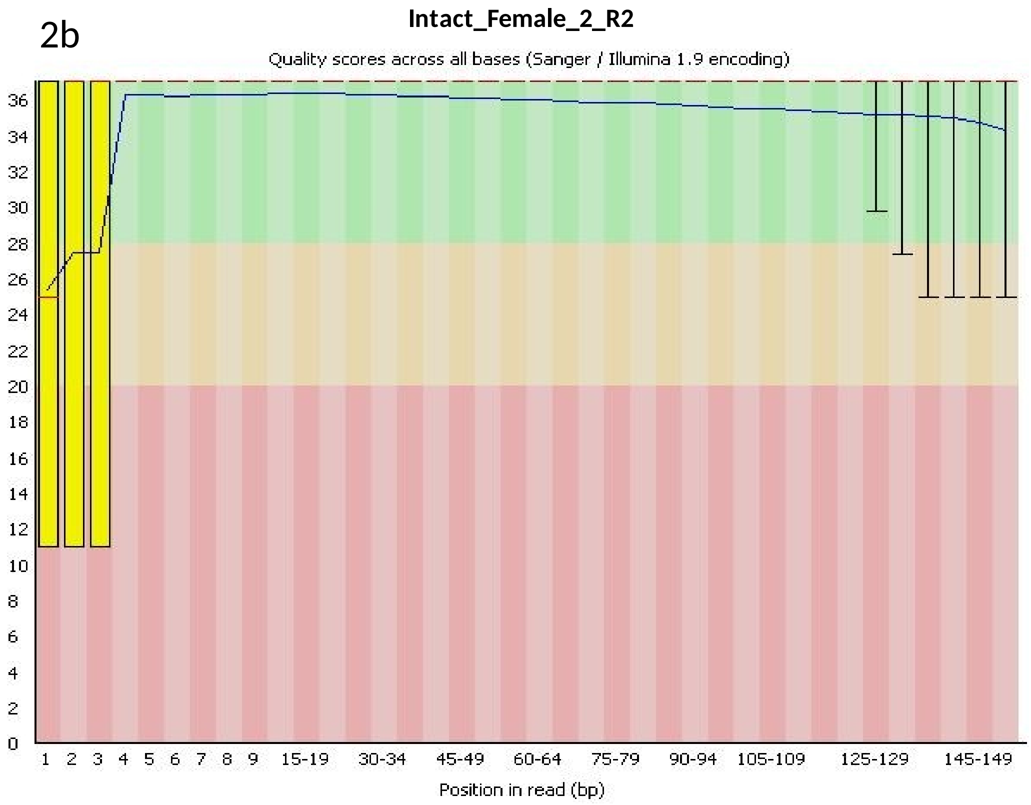

Intact_Female_2_R2

<u>Supplementary Figures 2a and 2b Intact Female #2</u>. FASTQ quality assessment.

Box and whisker plots illustrate the distribution of per base quality for left (R1; 2a) and right (R2; 2b) position reads. The blue line indicates mean value and the yellow box represents interquartile range (25%-75%). Upper and lower whiskers indicate 10% and 90% points respectively. Plots were generated by FASTQC program.

3a

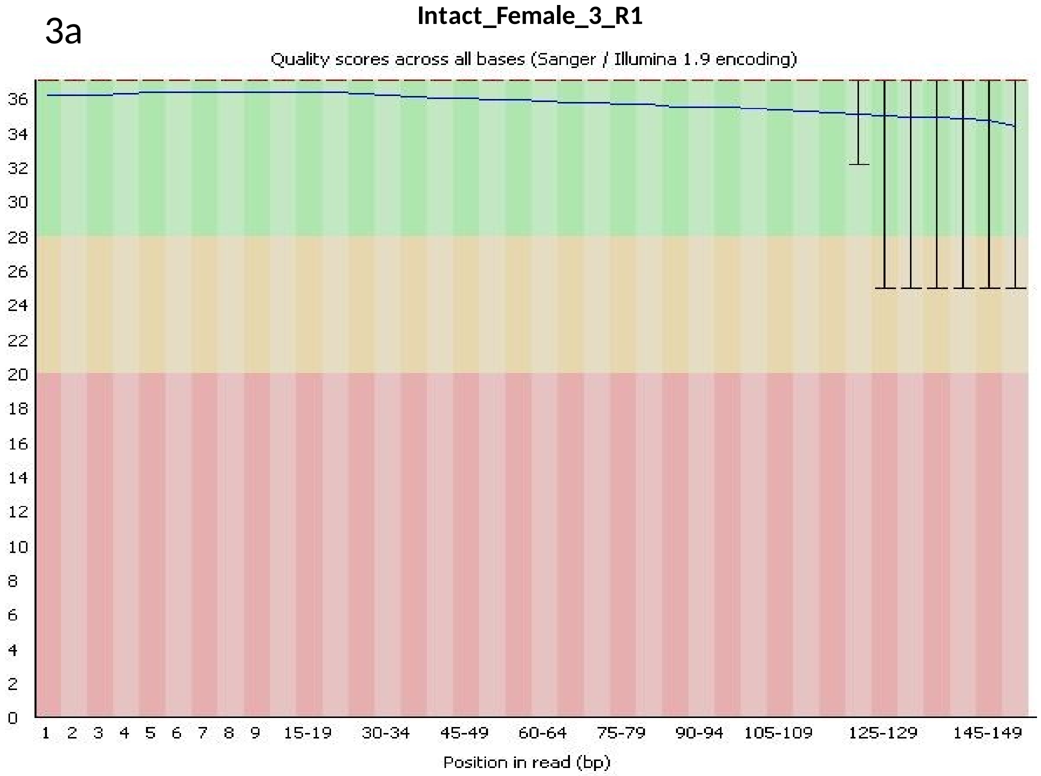

3b

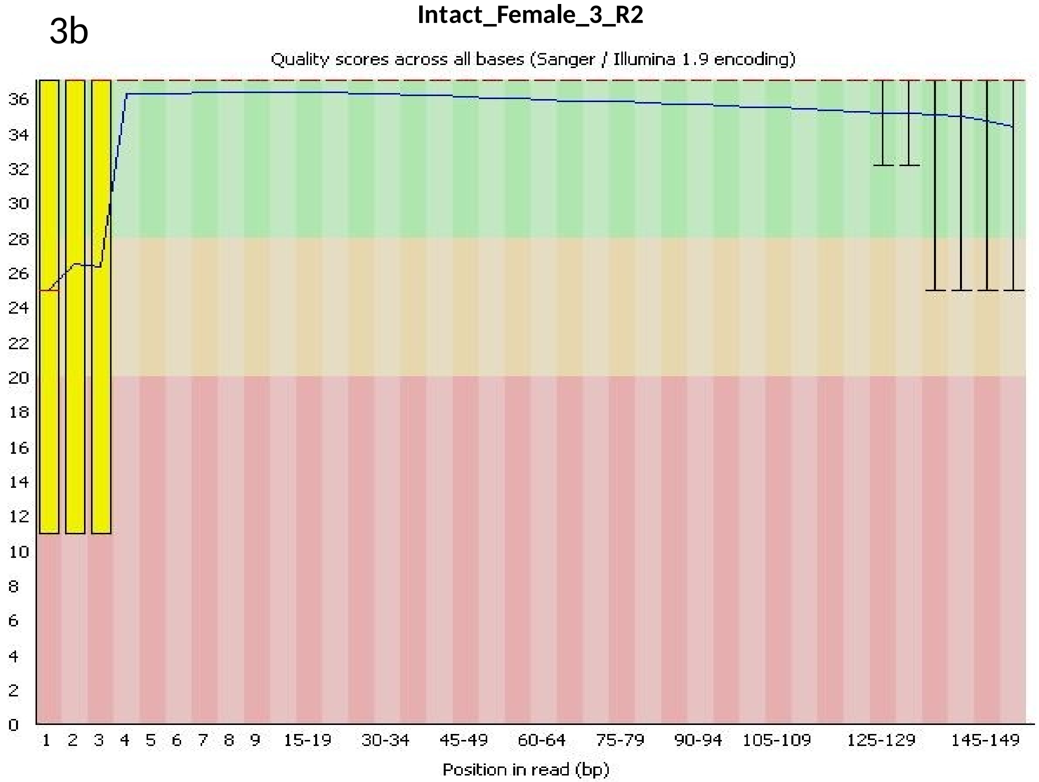

Intact_Female_3

Intact_Female_3_R2

<u>Supplementary Figures 3a and 3b Intact Female #3</u>. FASTQ quality assessment.

Box and whisker plots illustrate the distribution of per base quality for left (R1; 3a) and right (R2; 3b) position reads. The blue line indicates mean value and the yellow box represents interquartile range (25%-75%). Upper and lower whiskers indicate 10% and 90% points respectively. Plots were generated by FASTQC program.

4a

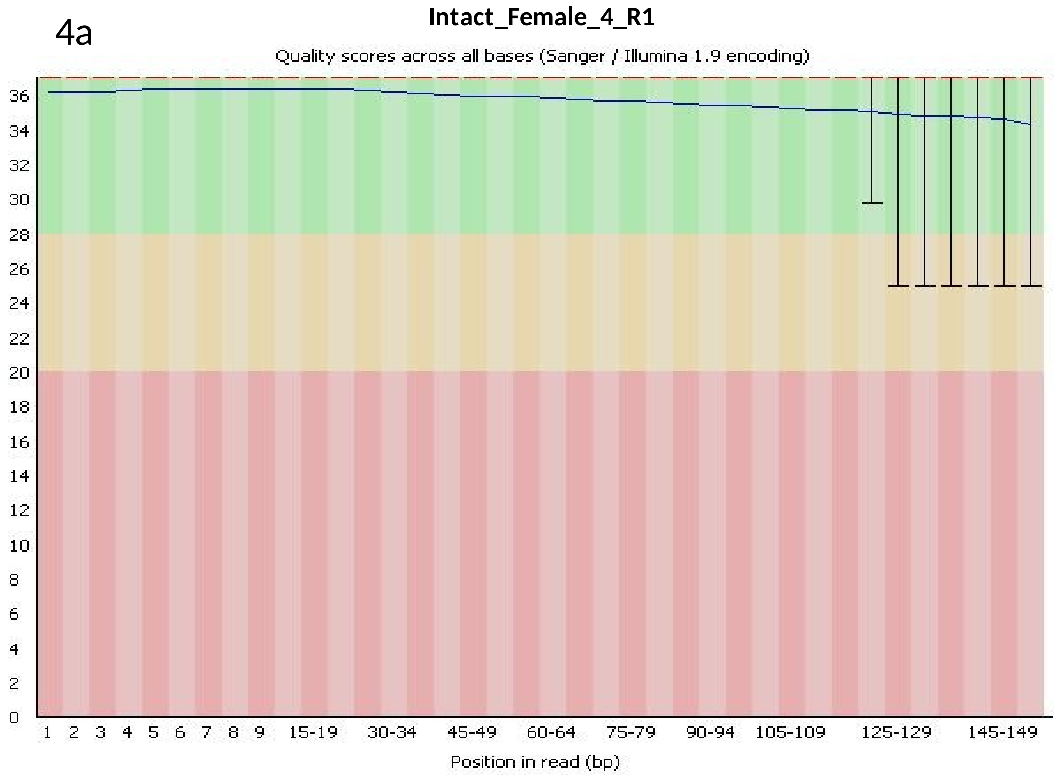

Intact_Female_4

4b

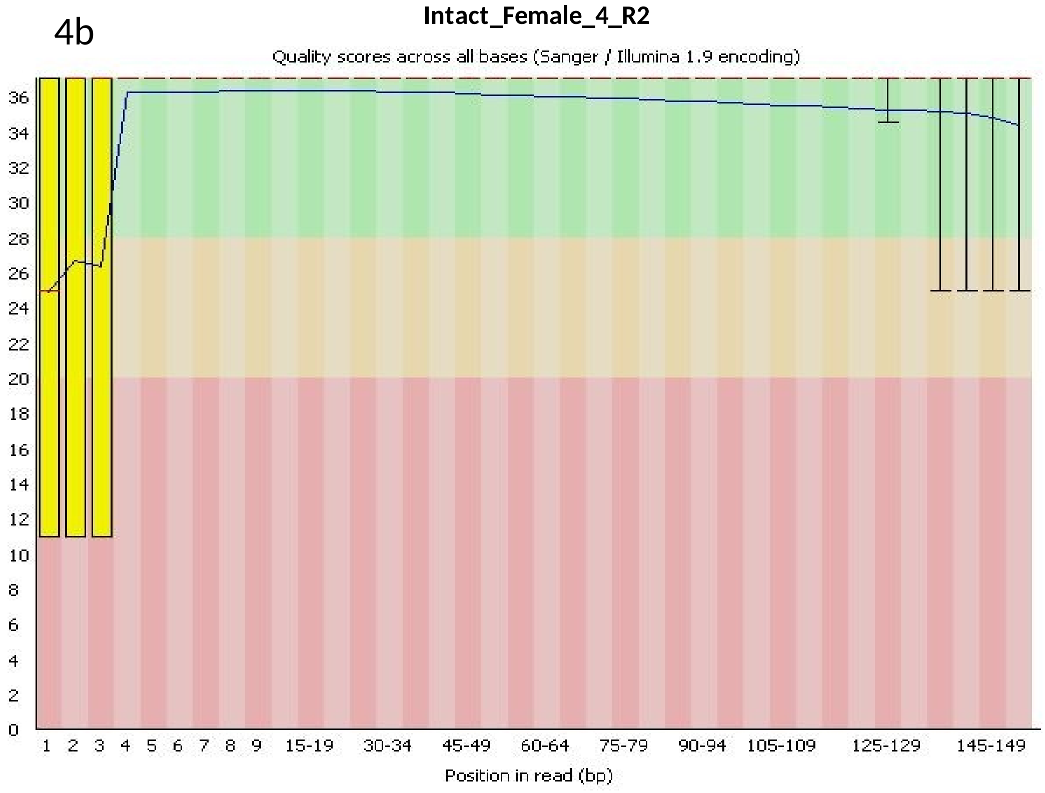

Intact_Female_4_R2

Supplementary Figures 4a and 4b Intact Female #4. FASTQ quality assessment.
Box and whisker plots illustrate the distribution of per base quality for left (R1; 4a) and right (R2; 4b) position reads. The blue line indicates mean value and the yellow box represents interquartile range (25%-75%). Upper and lower whiskers indicate 10% and 90% points respectively. Plots were generated by FASTQC program.

5a

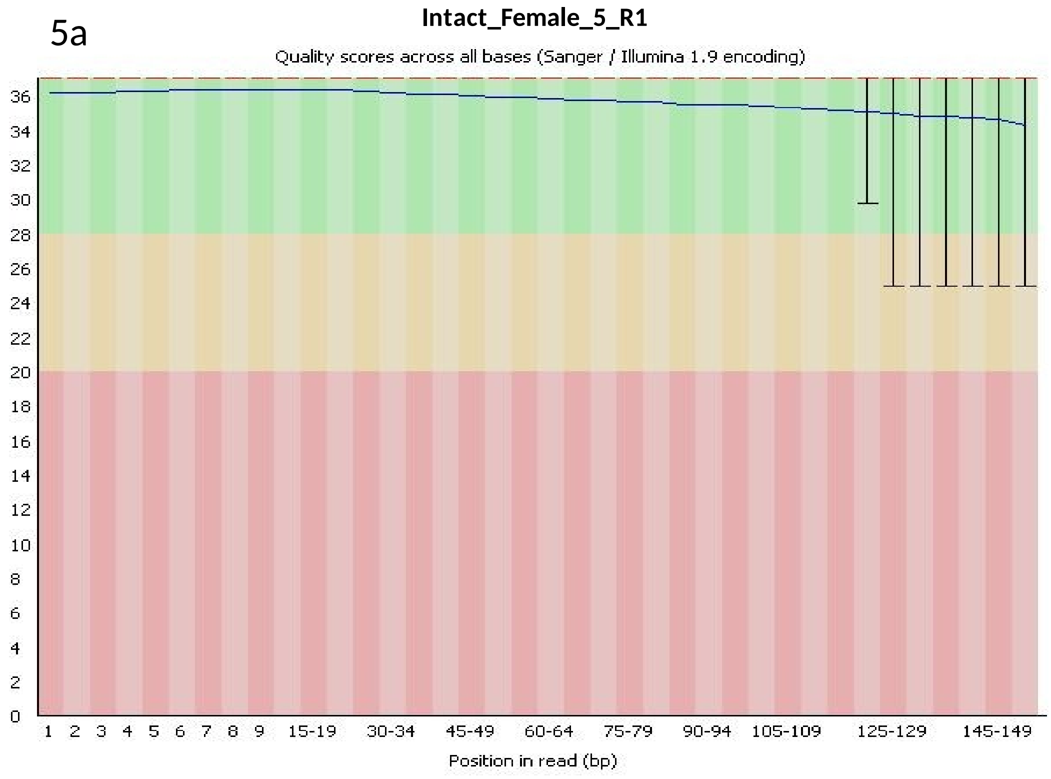

Intact_Female_5

5b

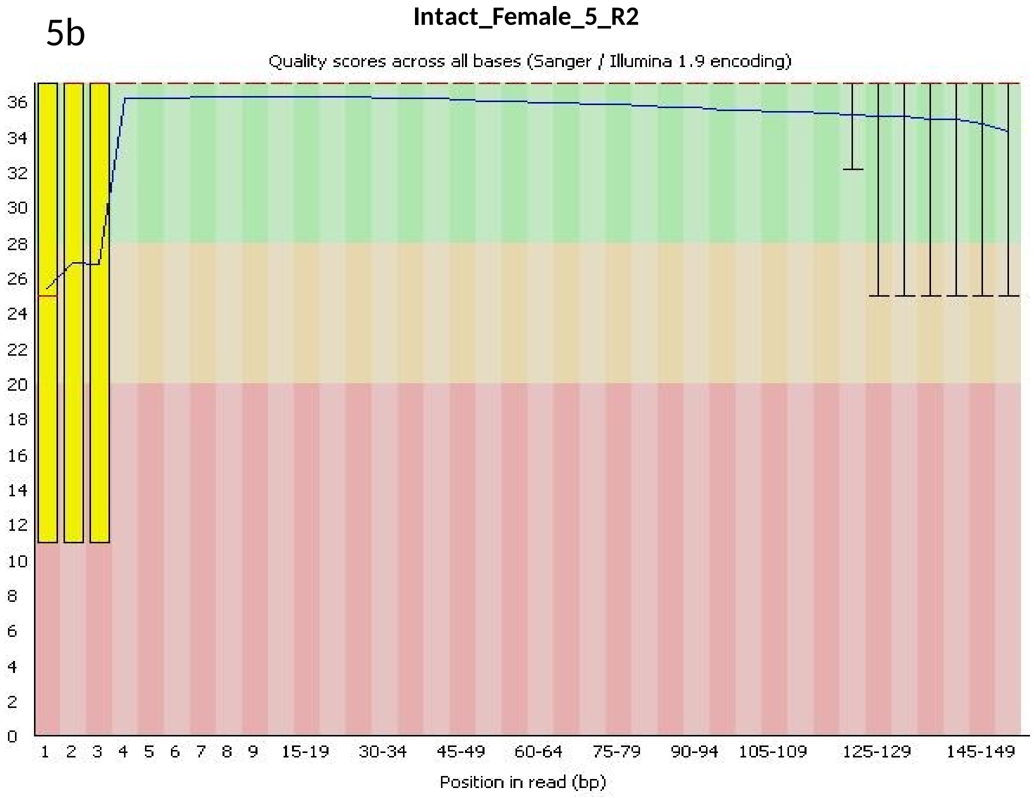

Intact_Female_5_R2

Supplementary Figures 5a and 5b Intact Female #5. FASTQ quality assessment.

Box and whisker plots illustrate the distribution of per base quality for left (R1; 5a) and right (R2; 5b) position reads. The blue line indicates mean value and the yellow box represents interquartile range (25%-75%). Upper and lower whiskers indicate 10% and 90% points respectively. Plots were generated by FASTQC program.

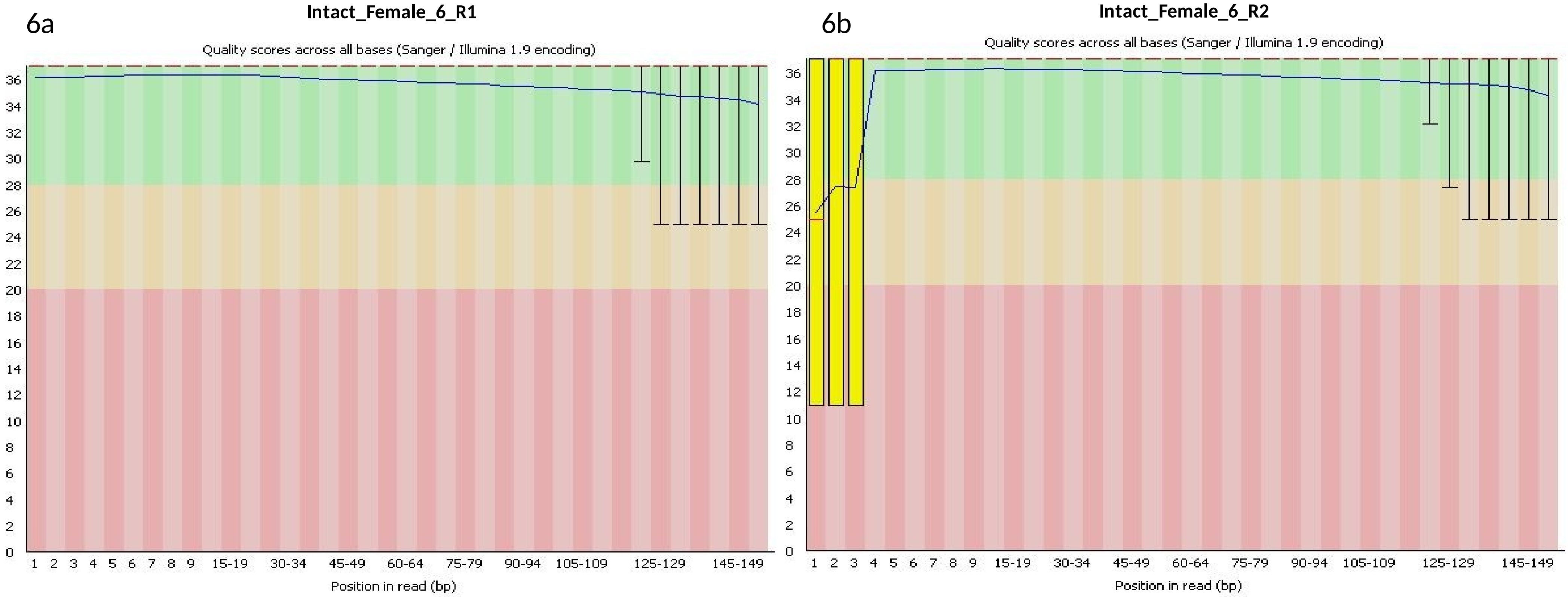

Intact_Female_6

Intact_Female_6_R2

<u>Supplementary Figures 6a and 6b Intact Female #6</u>. FASTQ quality assessment.

Box and whisker plots illustrate the distribution of per base quality for left (R1; 6a) and right (R2; 6b) position reads. The blue line indicates mean value and the yellow box represents interquartile range (25%-75%). Upper and lower whiskers indicate 10% and 90% points respectively. Plots were generated by FASTQC program.

7a

7b

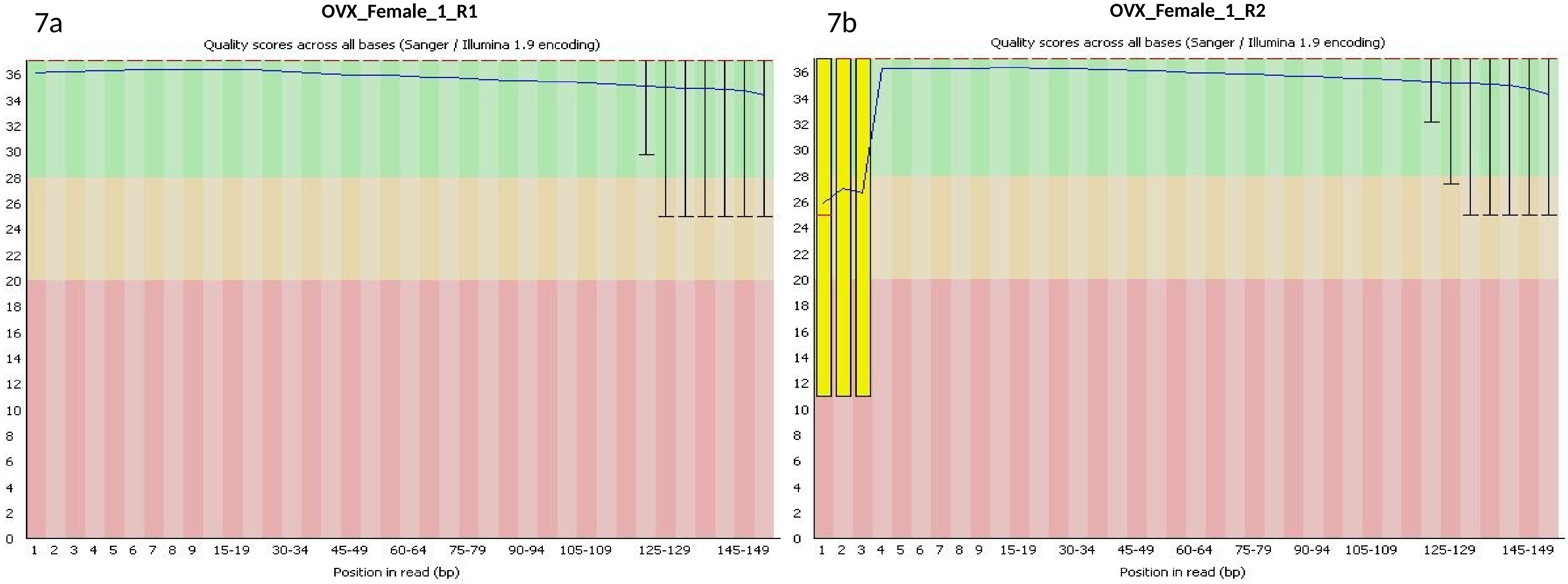

OVX_Female_1

OVX_Female_1_R2

Supplementary Figures 7a and 7b OVX Female #1. FASTQ quality assessment.
Box and whisker plots illustrate the distribution of per base quality for left (R1; 7a) and right (R2; 7b) position reads. The blue line indicates mean value and the yellow box represents interquartile range (25%-75%). Upper and lower whiskers indicate 10% and 90% points respectively. Plots were generated by FASTQC program.

8a

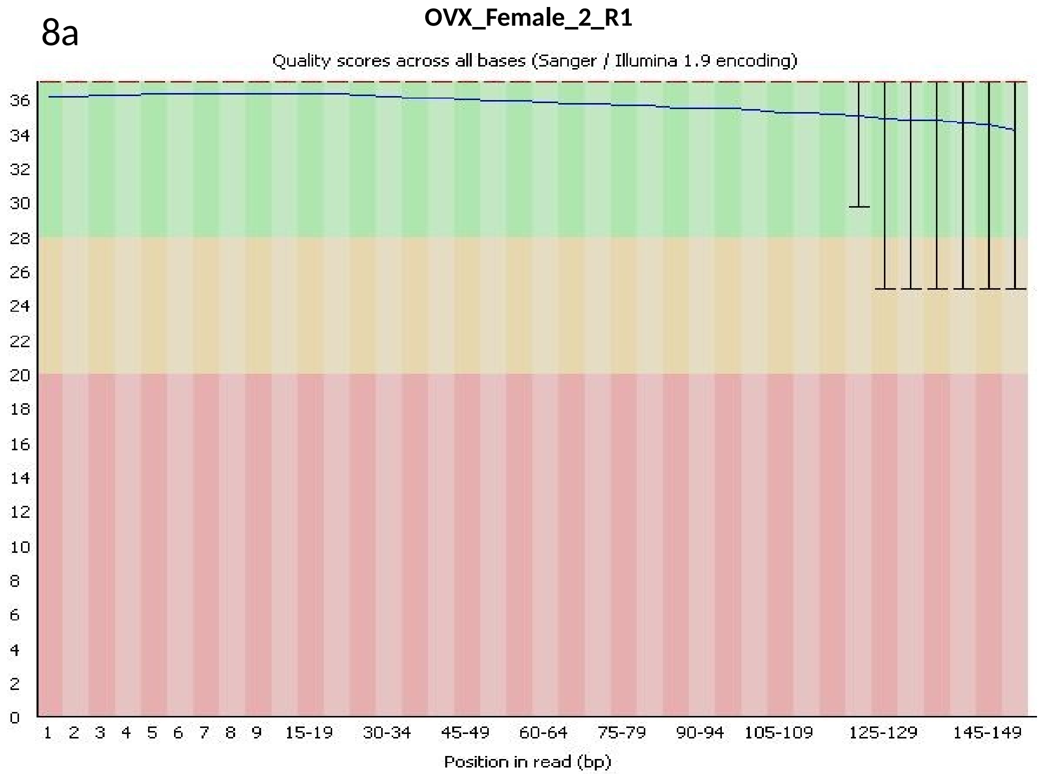

8b

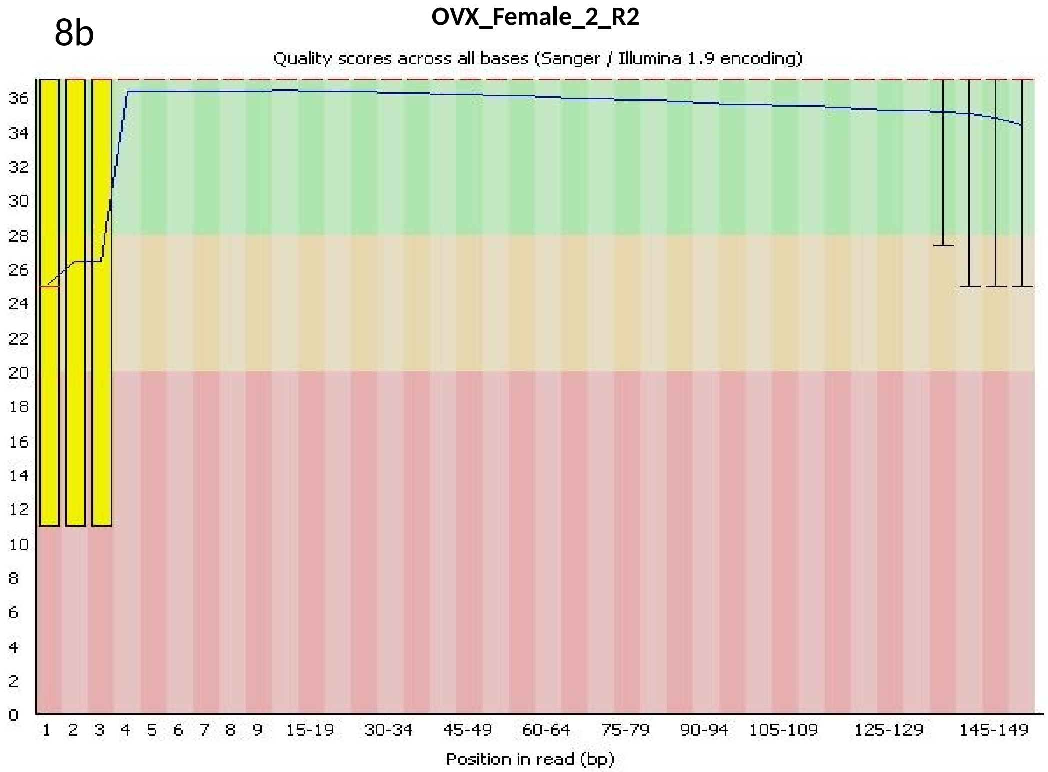

OVX_Female_2

OVX_Female_2_R2

Supplementary Figures 8a and 8b OVX Female #2. FASTQ quality assessment.

Box and whisker plots illustrate the distribution of per base quality for left (R1; 8a) and right (R2; 8b) position reads. The blue line indicates mean value and the yellow box represents interquartile range (25%-75%). Upper and lower whiskers indicate 10% and 90% points respectively. Plots were generated by FASTQC program.

9a

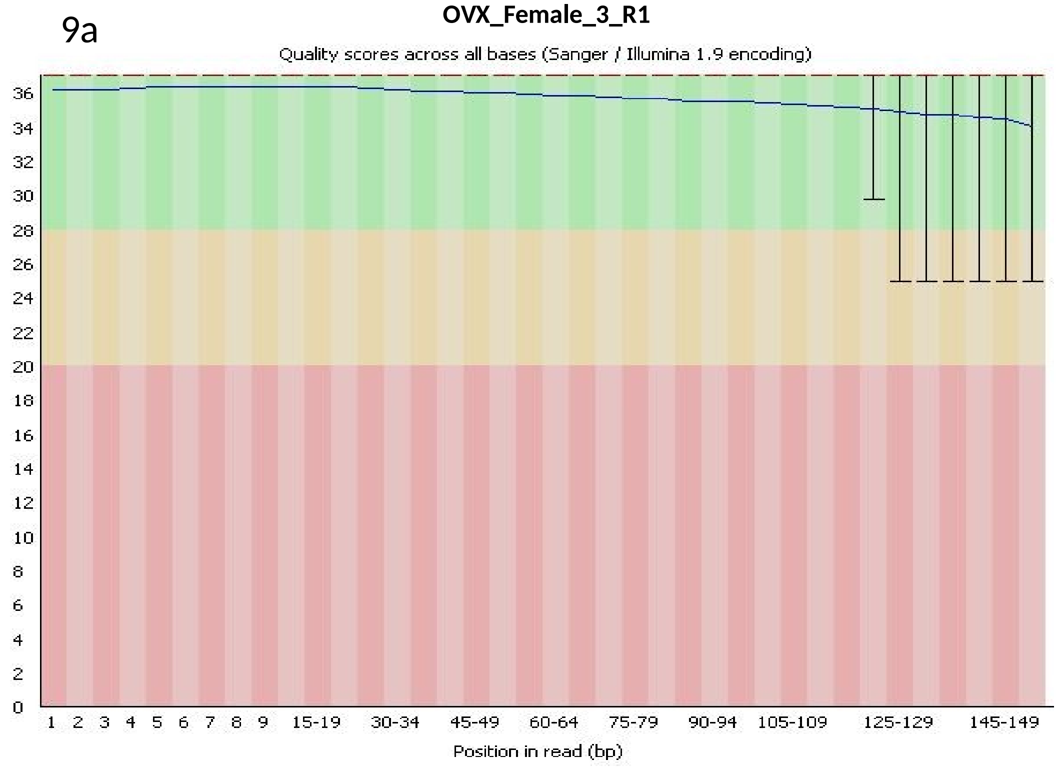

9b

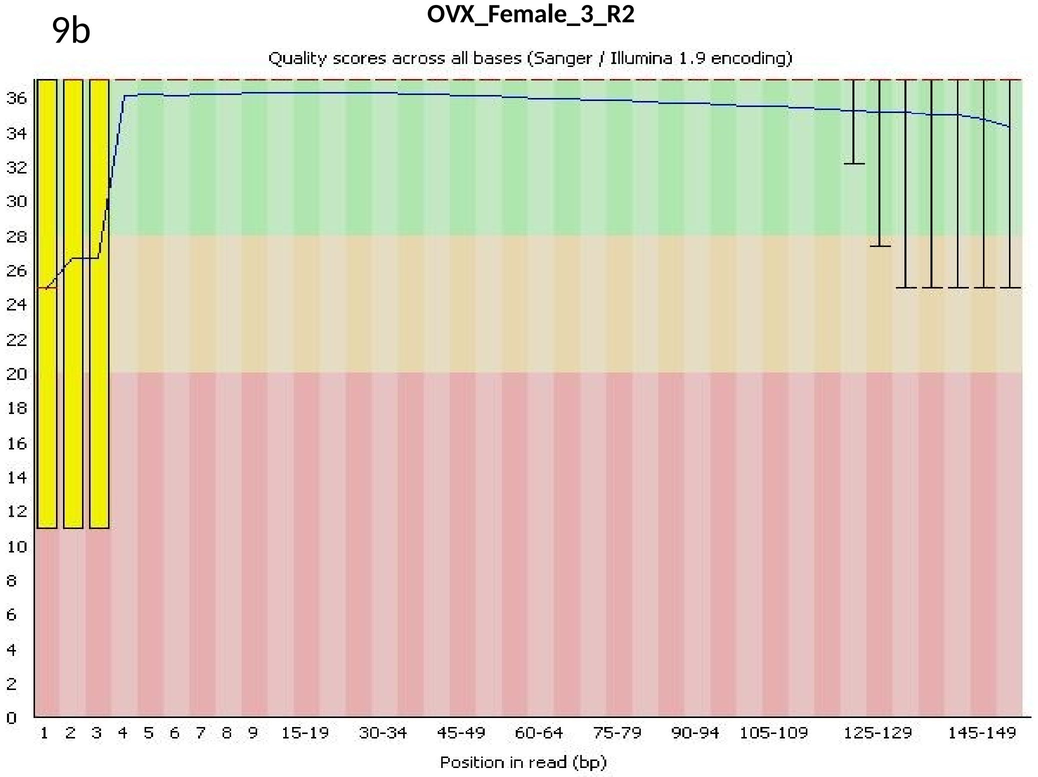

OVX_Female_3

OVX_Female_3_R2

<u>Supplementary Figures 9a and 9b OVX Female #3</u>. FASTQ quality assessment.

Box and whisker plots illustrate the distribution of per base quality for left (R1; 9a) and right (R2; 9b) position reads. The blue line indicates mean value and the yellow box represents interquartile range (25%-75%). Upper and lower whiskers indicate 10% and 90% points respectively. Plots were generated by FASTQC program.

10a

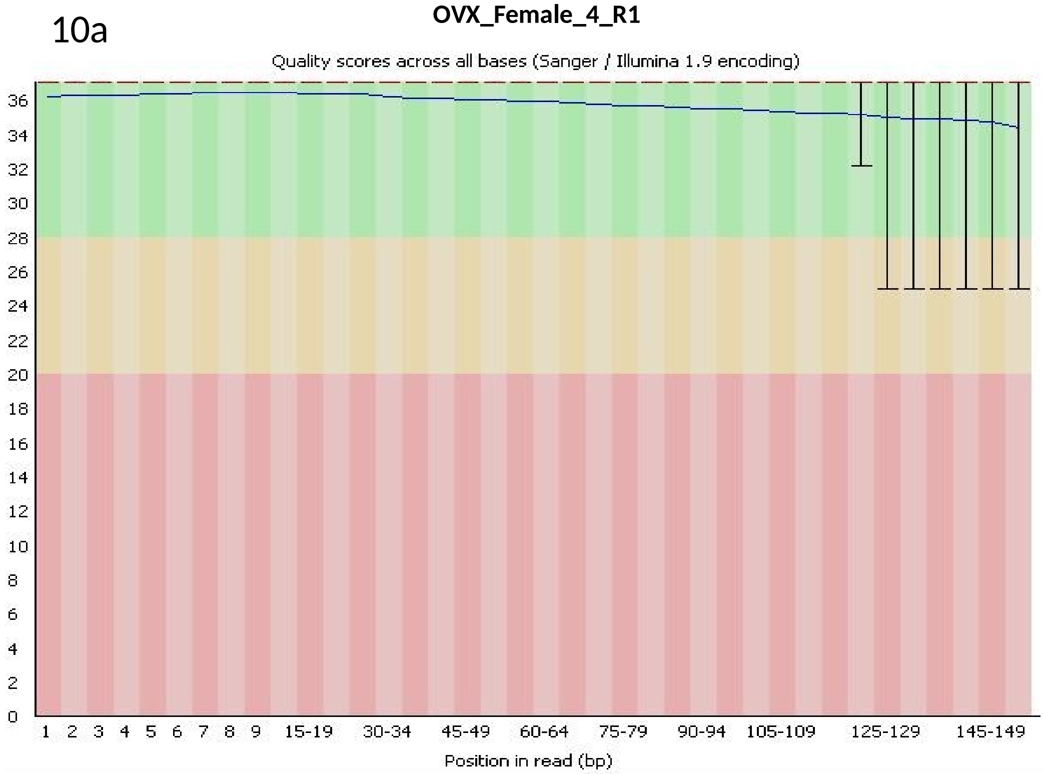

OVX_Female_4

10b

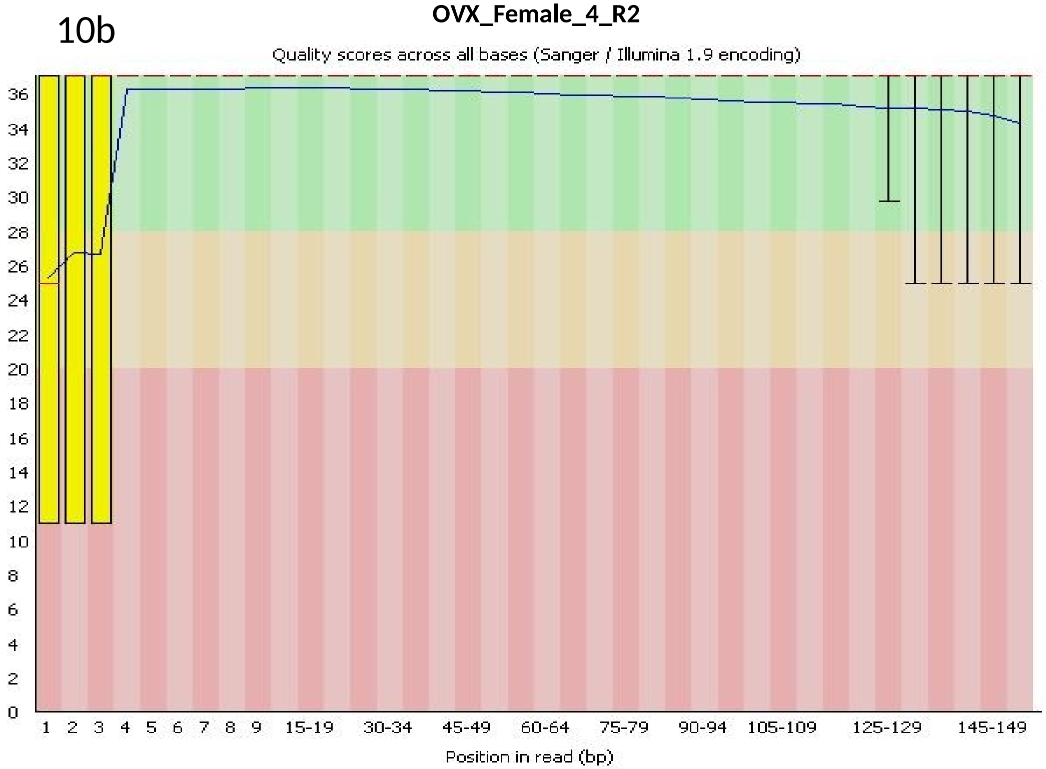

OVX_Female_4_R2

<u>Supplementary Figures 10a and 10b OVX Female #4</u>. FASTQ quality assessment.
Box and whisker plots illustrate the distribution of per base quality for left (R1; 10a) and right (R2; 10b) position reads. The blue line indicates mean value and the yellow box represents interquartile range (25%-75%). Upper and lower whiskers indicate 10% and 90% points respectively. Plots were generated by FASTQC program.

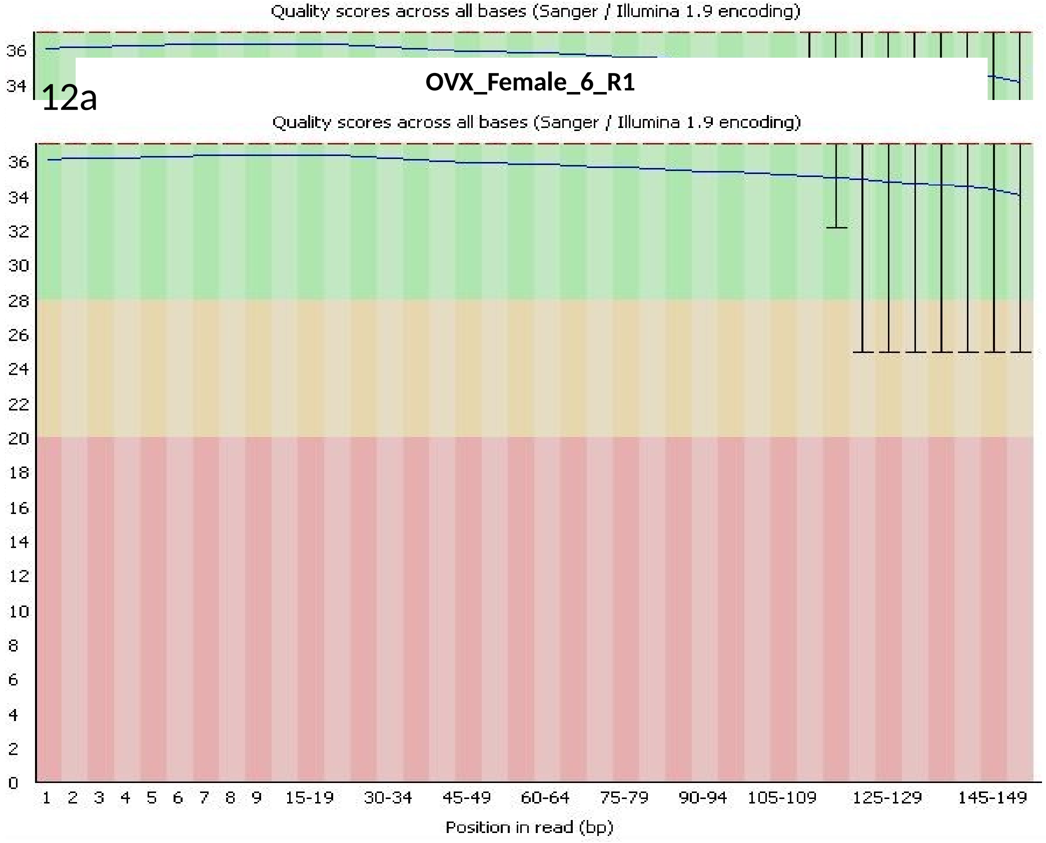

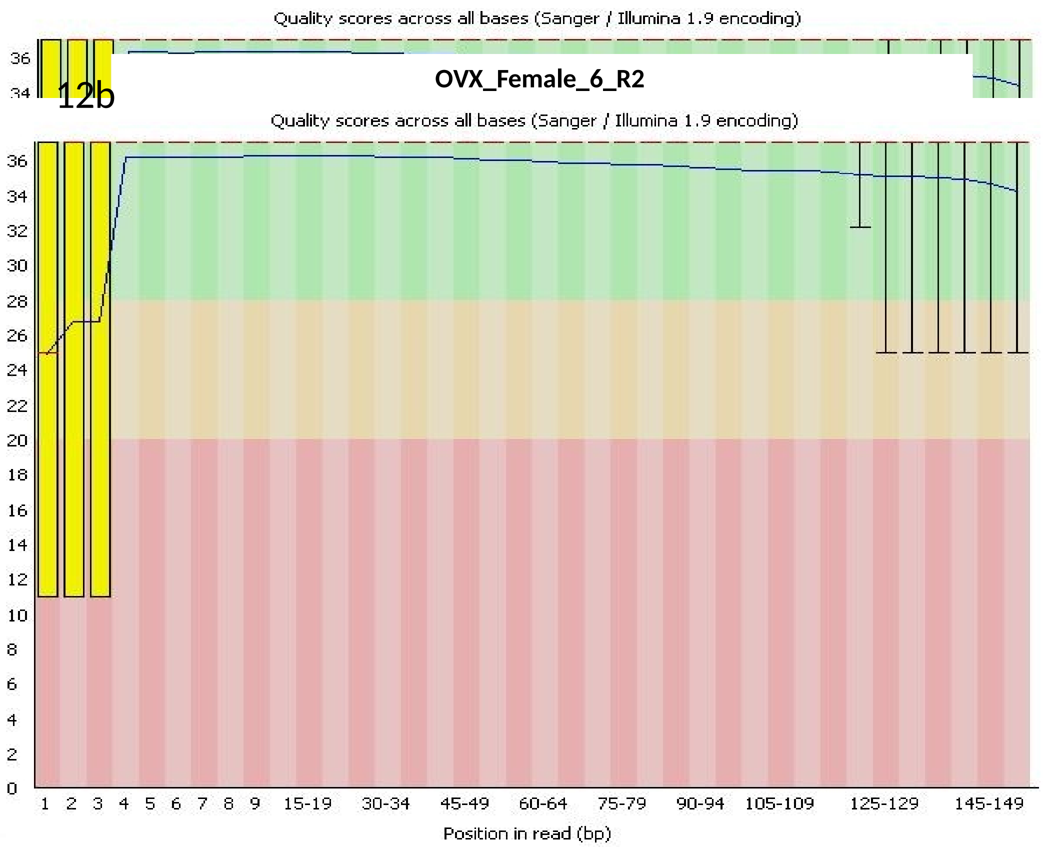

OVX_Female_6 OVX_Female_6_R2

<u>Supplementary Figures 11a and 11b OVX Female #5</u>. FASTQ quality assessment.
Box and whisker plots illustrate the distribution of per base quality for left (R1; 11a) and right (R2; 11b) position reads. The blue line indicates mean value and the yellow box represents interquartile range (25%-75%). Upper and lower whiskers indicate 10% and 90% points respectively. Plots were generated by FASTQC program.

<u>Supplementary Figures 12a and 12b OVX Female #6</u>. FASTQ quality assessment.
Box and whisker plots illustrate the distribution of per base quality for left (R1; 12a) and right (R2; 12b) position reads. The blue line indicates mean value and the yellow box represents interquartile range (25%-75%). Upper and lower whiskers indicate 10% and 90% points respectively. Plots were generated by FASTQC program.

13a

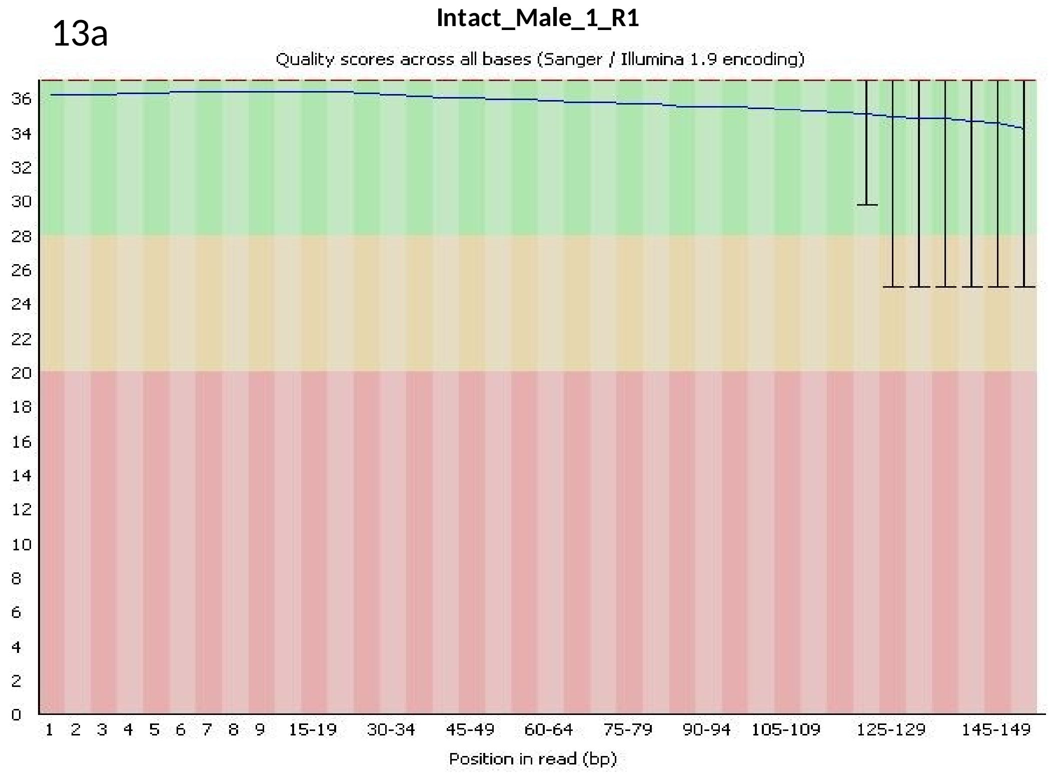

13b

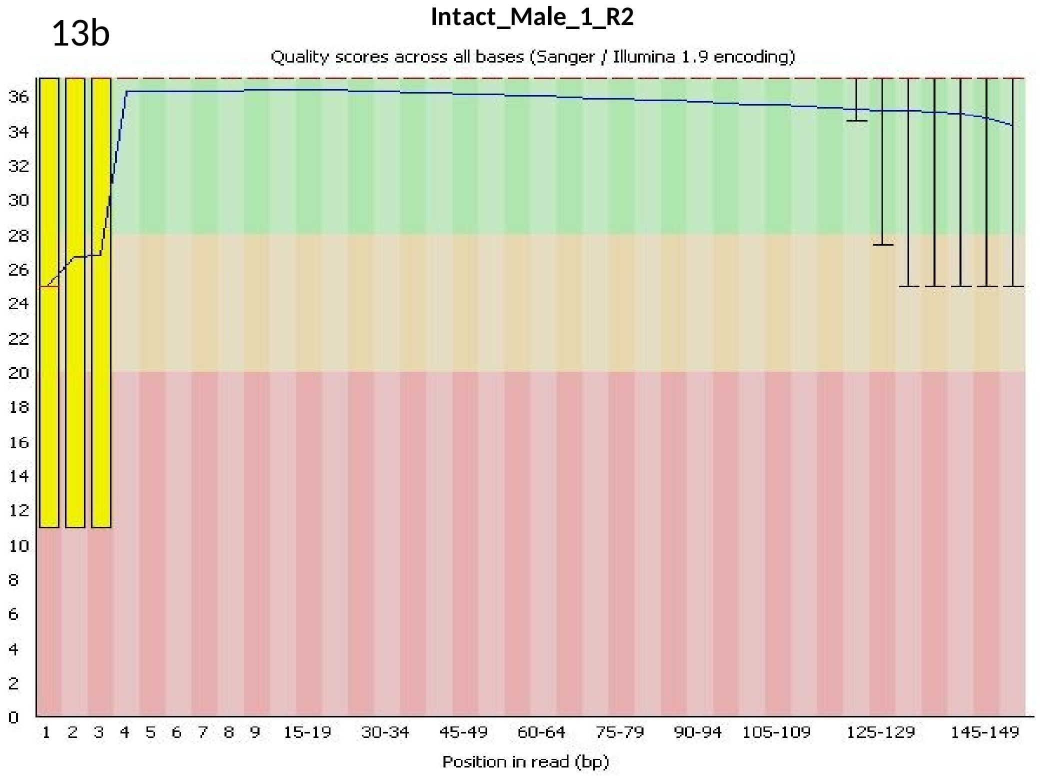

Intact_Male_1

Intact_Male_1_R2

Supplementary Figures 13a and 13b Intact Male #1. FASTQ quality assessment.
Box and whisker plots illustrate the distribution of per base quality for left (R1; 13a) and right (R2; 13b) position reads. The blue line indicates mean value and the yellow box represents interquartile range (25%-75%). Upper and lower whiskers indicate 10% and 90% points respectively. Plots were generated by FASTQC program.

14a

Intact_Male_2_R1

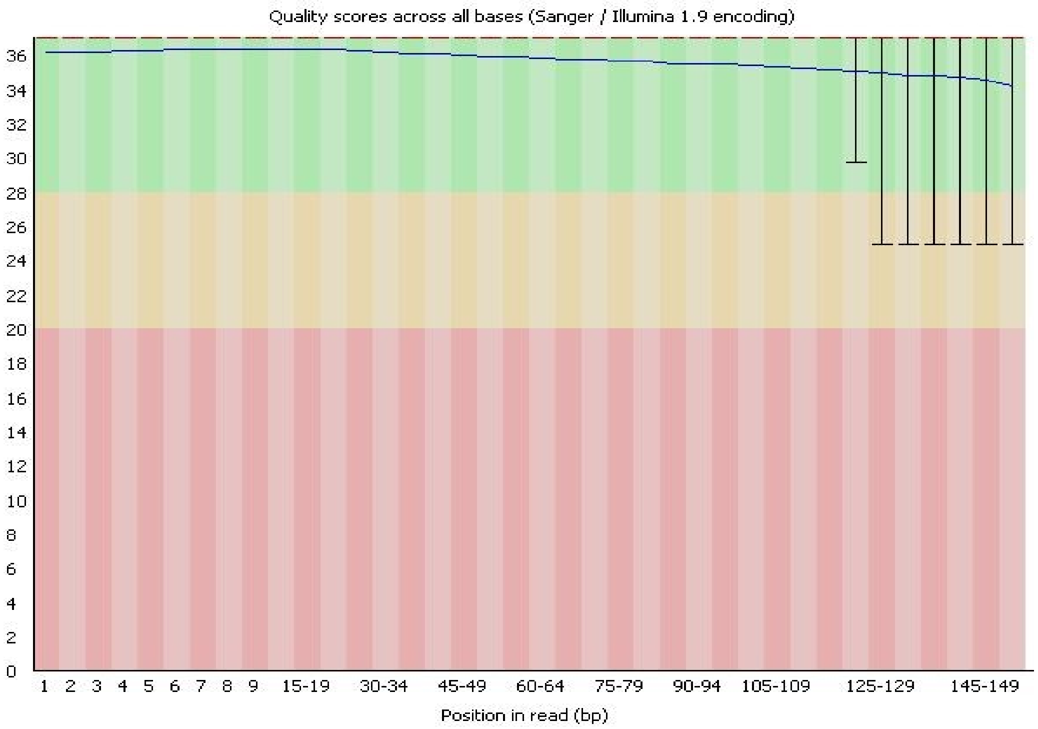

14b

Intact_Male_2_R2

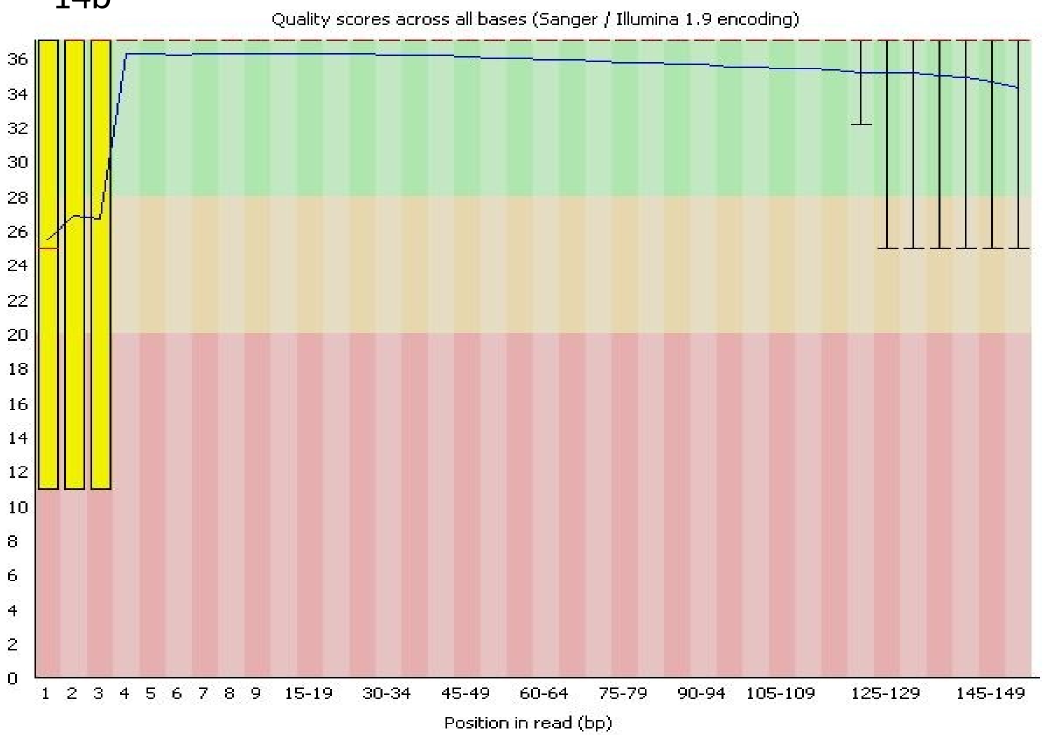

Intact_Male_2

Intact_Male_2_R2

<u>Supplementary Figures 14a and 14b Intact Male #2</u>. FASTQ quality assessment.

Box and whisker plots illustrate the distribution of per base quality for left (R1; 14a) and right (R2; 14b) position reads. The blue line indicates mean value and the yellow box represents interquartile range (25%-75%). Upper and lower whiskers indicate 10% and 90% points respectively. Plots were generated by FASTQC program.

15a

Intact_Male_3_R1

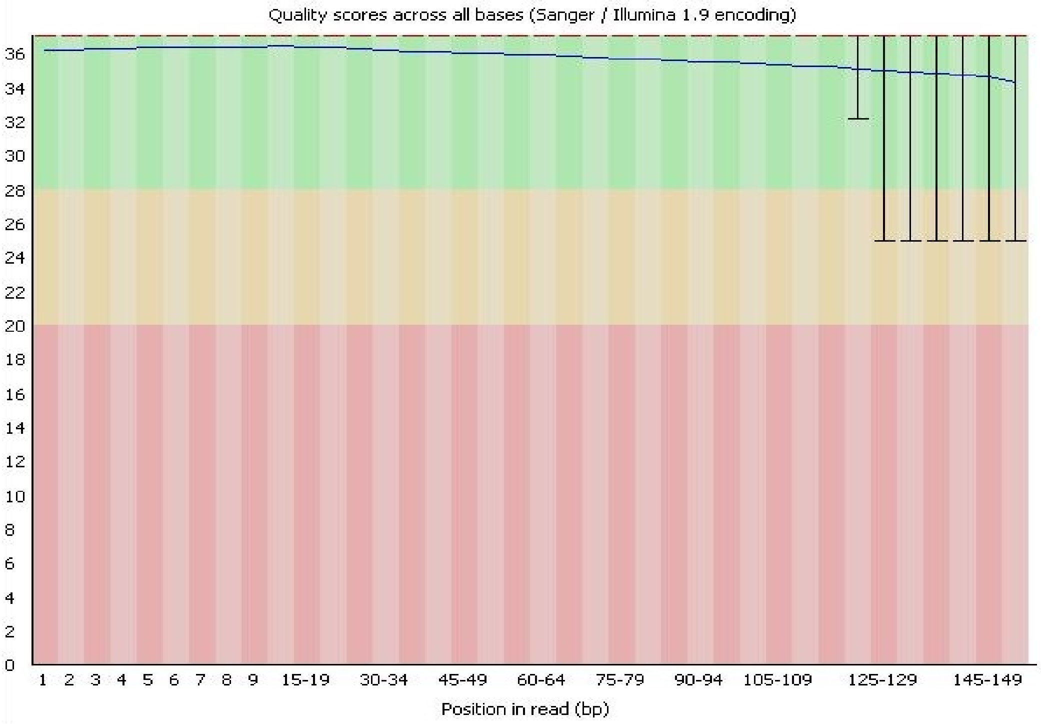

Intact_Male_3

15b

Intact_Male_3_R2

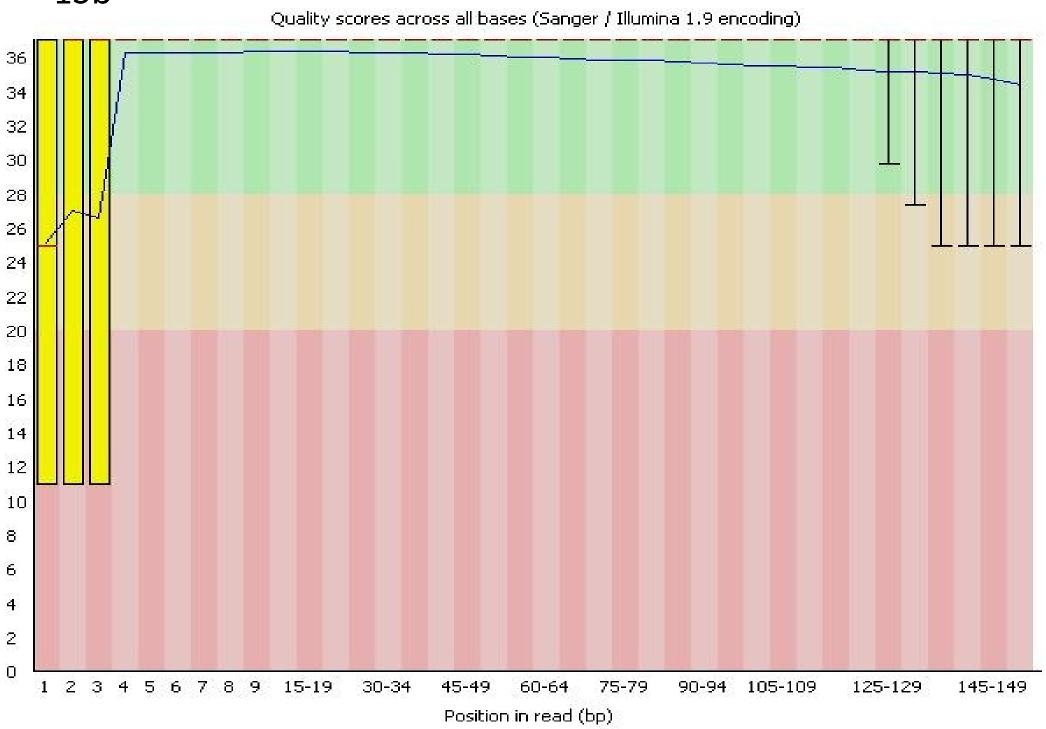

Intact_Male_3_R2

<u>Supplementary Figures 15a and 15b Intact Male #3</u>. FASTQ quality assessment.
Box and whisker plots illustrate the distribution of per base quality for left (R1; 15a) and right (R2; 15b) position reads. The blue line indicates mean value and the yellow box represents interquartile range (25%-75%). Upper and lower whiskers indicate 10% and 90% points respectively. Plots were generated by FASTQC program.

16a

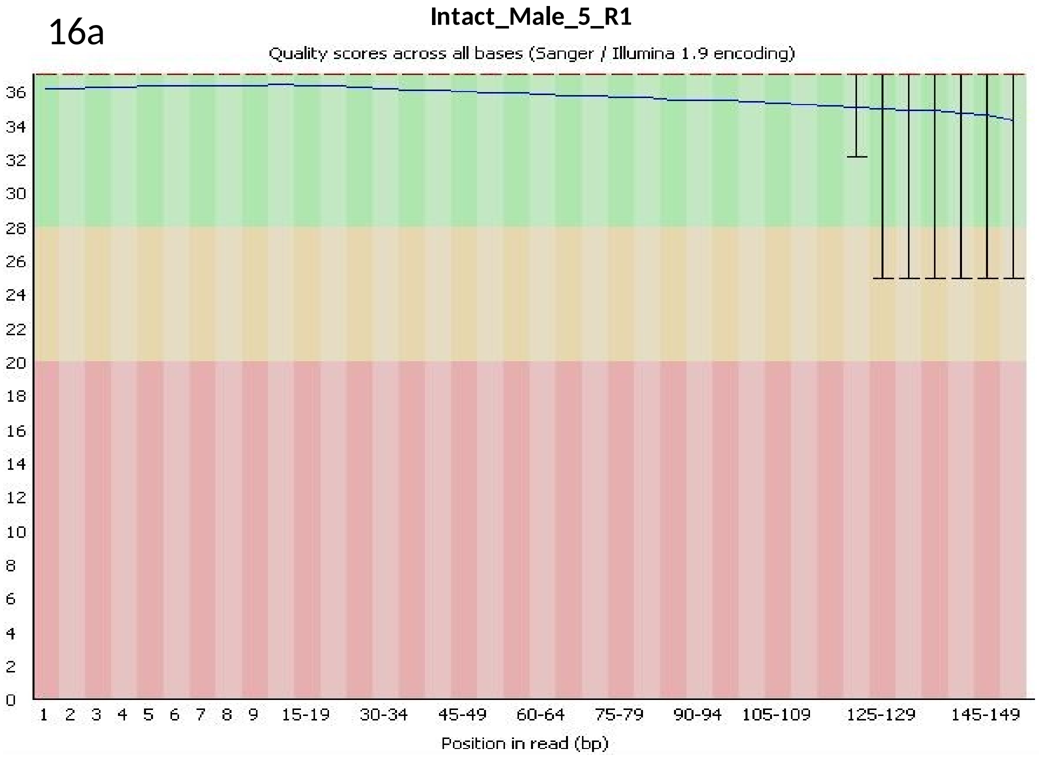

Intact_Male_5

16b

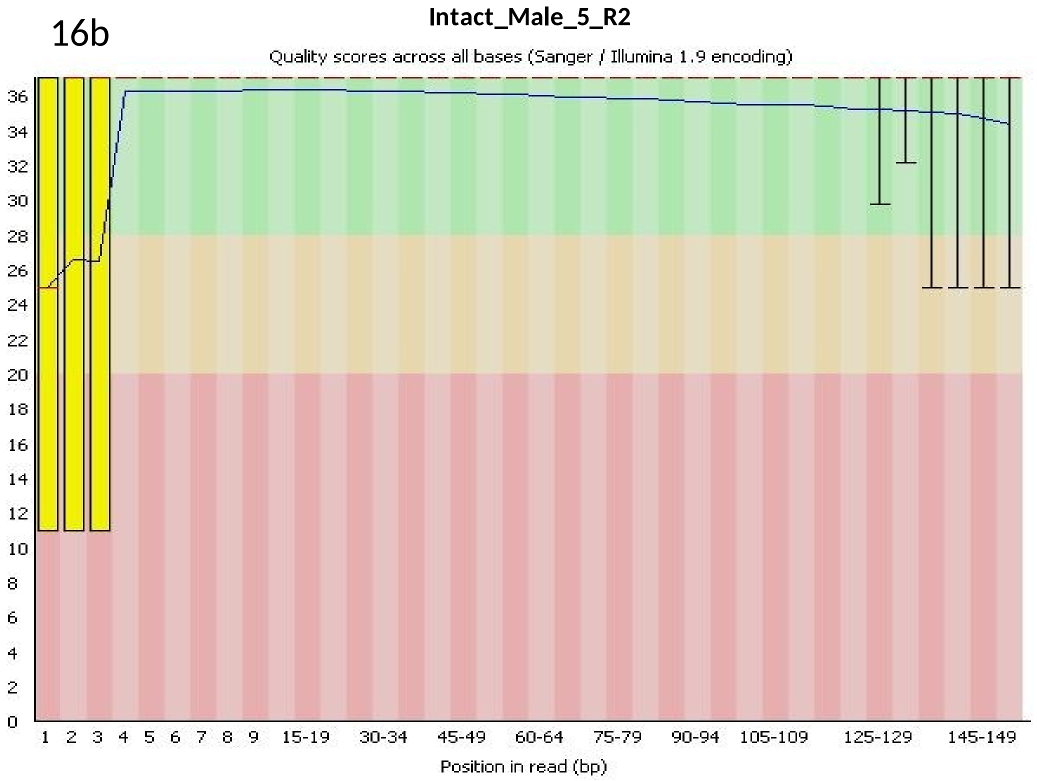

Intact_Male_5_R2

Supplementary Figures 16a and 16b Intact Male #5. FASTQ quality assessment.
Box and whisker plots illustrate the distribution of per base quality for left (R1; 16a) and right (R2; 16b) position reads. The blue line indicates mean value and the yellow box represents interquartile range (25%-75%). Upper and lower whiskers indicate 10% and 90% points respectively. Plots were generated by FASTQC program.

17a

17b

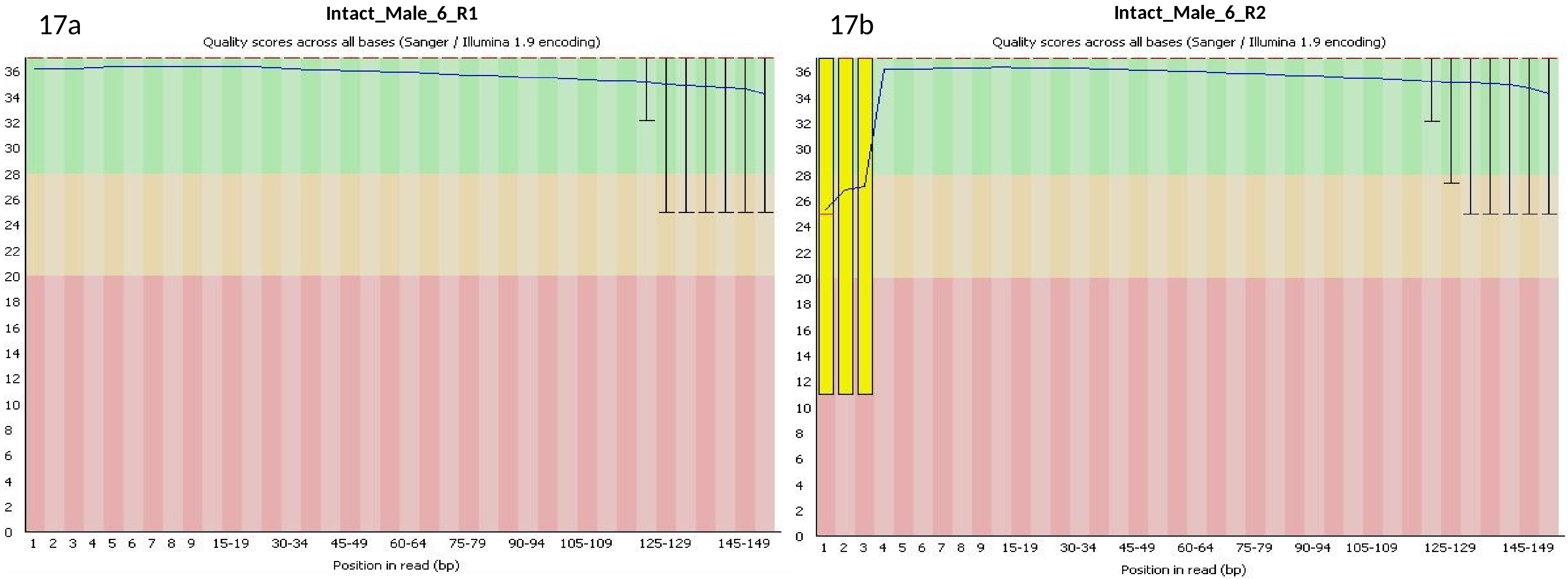

Intact_Male_6

Intact_Male_6_R2

Supplementary Figures 17a and 17b Intact Male #6. FASTQ quality assessment.
Box and whisker plots illustrate the distribution of per base quality for left (R1; 17a) and right (R2; 17b) position reads. The blue line indicates mean value and the yellow box represents interquartile range (25%-75%). Upper and lower whiskers indicate 10% and 90% points respectively. Plots were generated by FASTQC program.

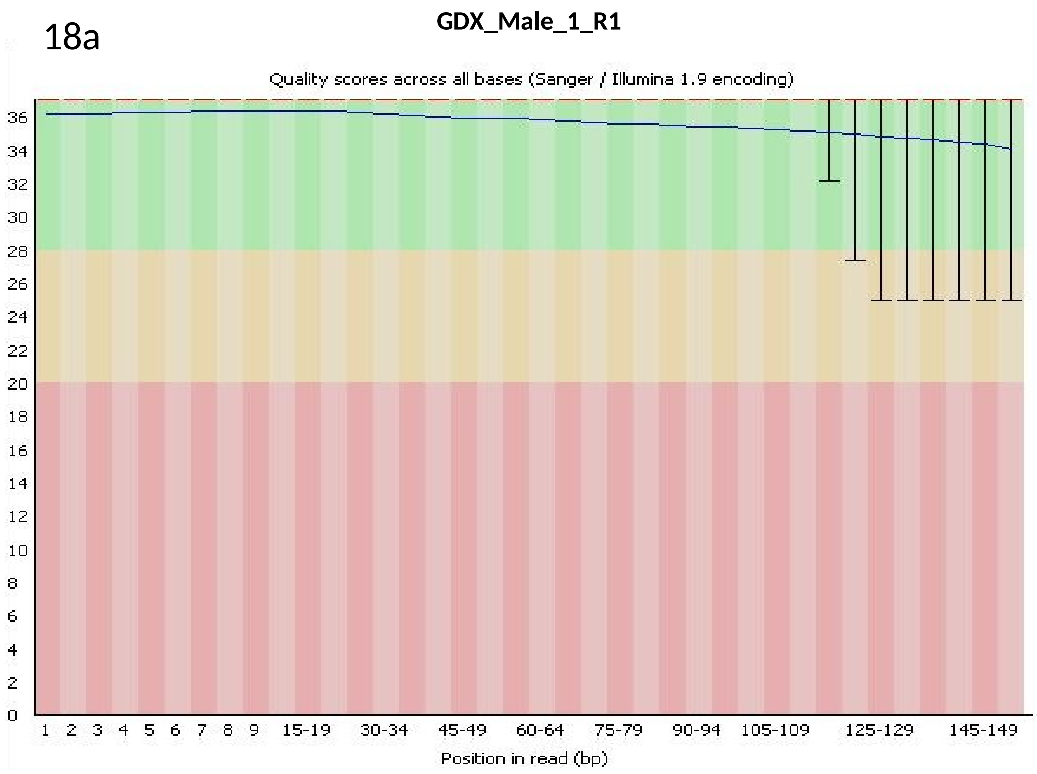

GDX_Male_1

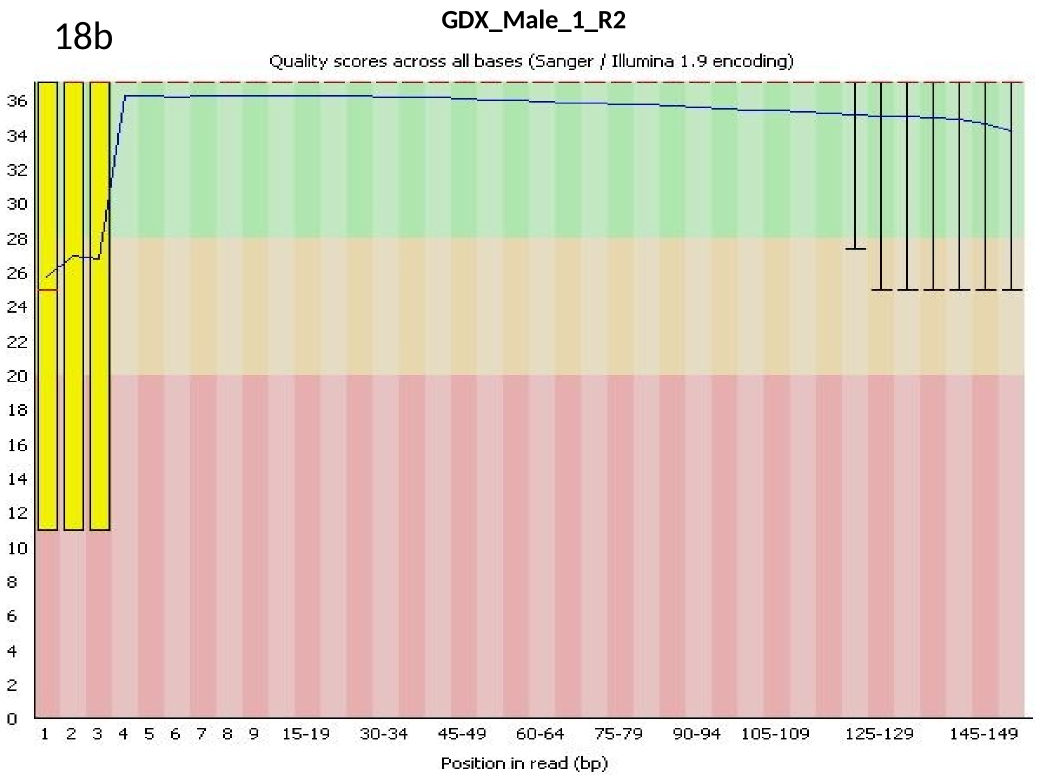

GDX_Male_1_R2

Supplementary Figures 18a and 18b GDX Male #1. FASTQ quality assessment.
Box and whisker plots illustrate the distribution of per base quality for left (R1; 18a) and right (R2; 18b) position reads. The blue line indicates mean value and the yellow box represents interquartile range (25%-75%). Upper and lower whiskers indicate 10% and 90% points respectively. Plots were generated by FASTQC program.

19a

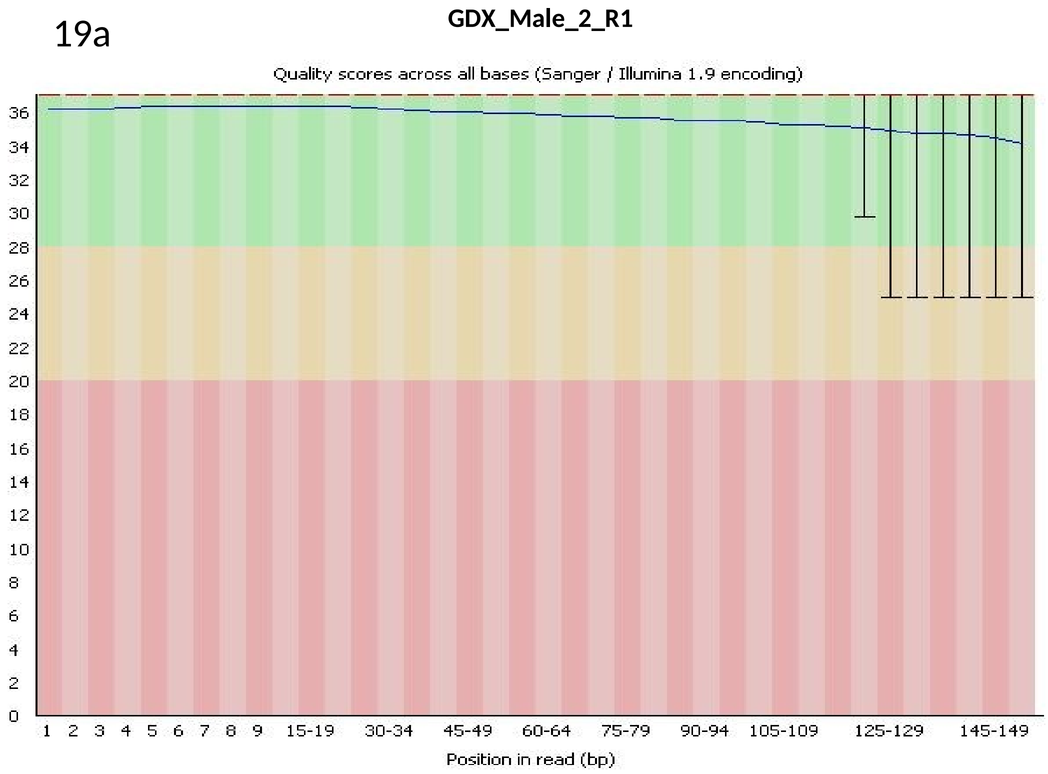

19b

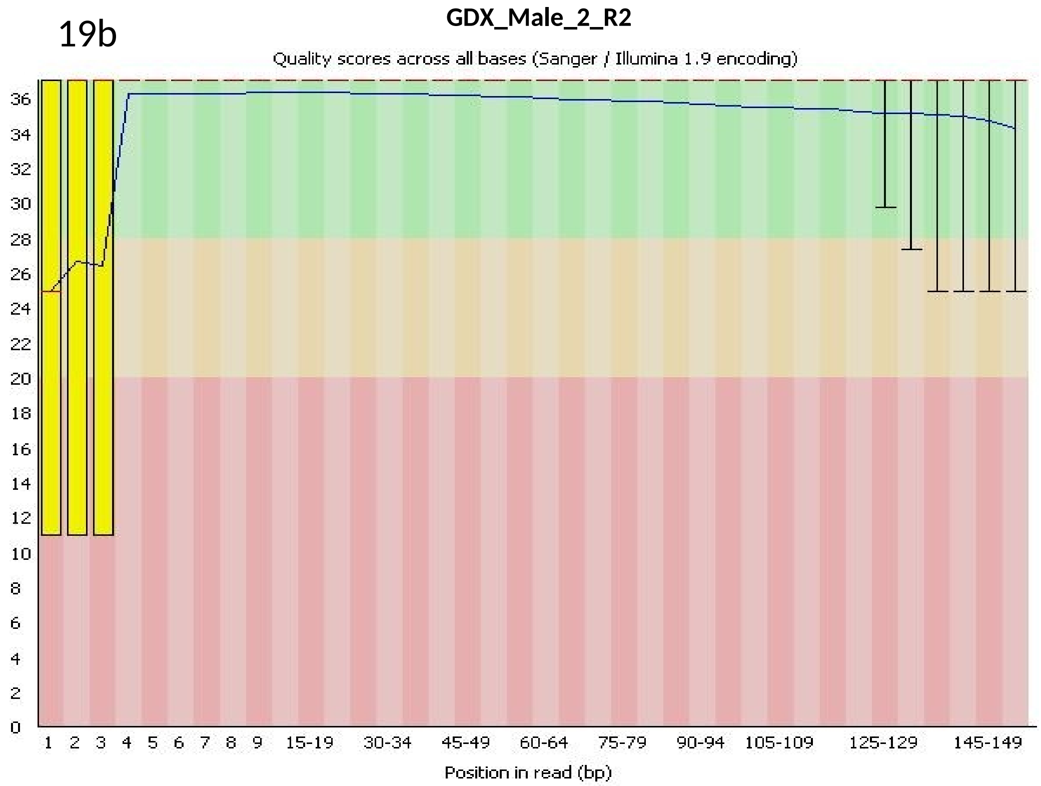

GDX_Male_2

GDX_Male_2_R2

Supplementary Figures 19a and 19b GDX Male #2. FASTQ quality assessment.

Box and whisker plots illustrate the distribution of per base quality for left (R1; 19a) and right (R2; 19b) position reads. The blue line indicates mean value and the yellow box represents interquartile range (25%-75%). Upper and lower whiskers indicate 10% and 90% points respectively. Plots were generated by FASTQC program.

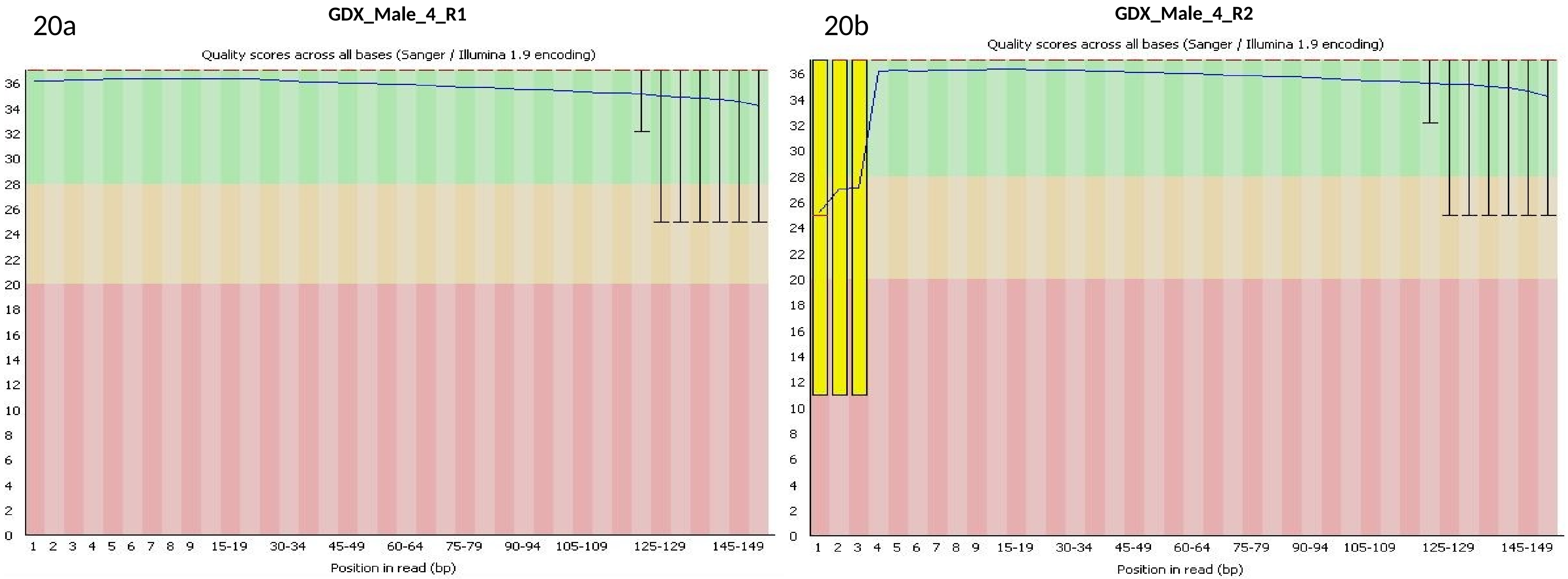

GDX_Male_4

GDX_Male_4_R2

Supplementary Figures 20a and 20b GDX Male #4. FASTQ quality assessment.
Box and whisker plots illustrate the distribution of per base quality for left (R1; 20a) and right (R2; 20b) position reads. The blue line indicates mean value and the yellow box represents interquartile range (25%-75%). Upper and lower whiskers indicate 10% and 90% points respectively. Plots were generated by FASTQC program.

21a

GDX_Male_5_R1

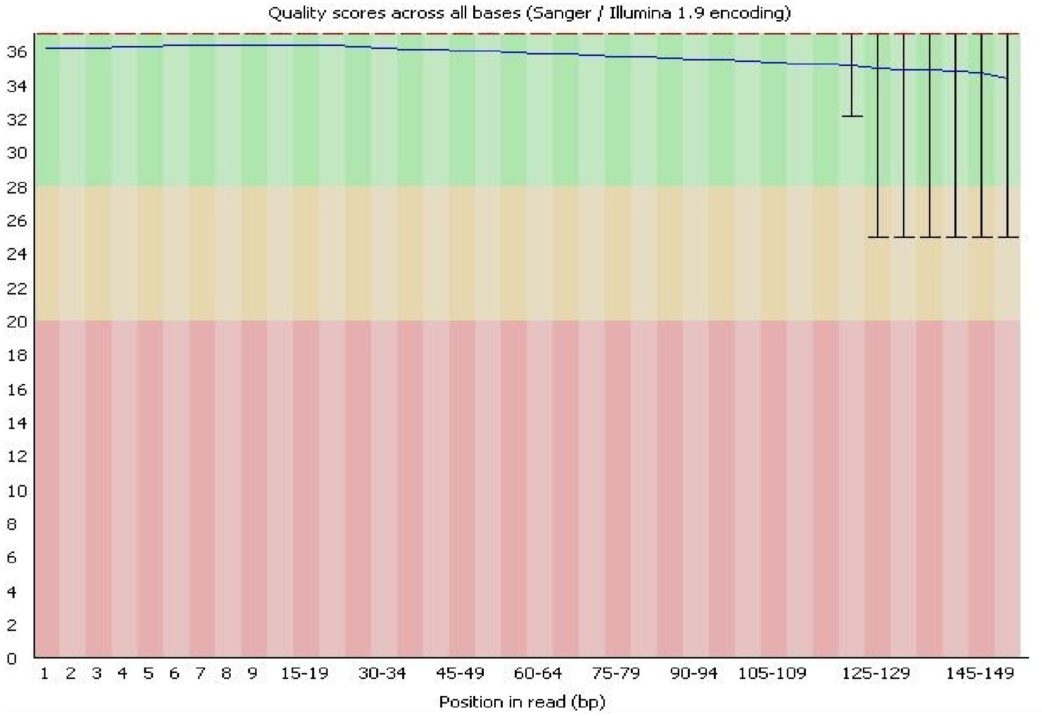

GDX_Male_5

21b

GDX_Male_5_R2

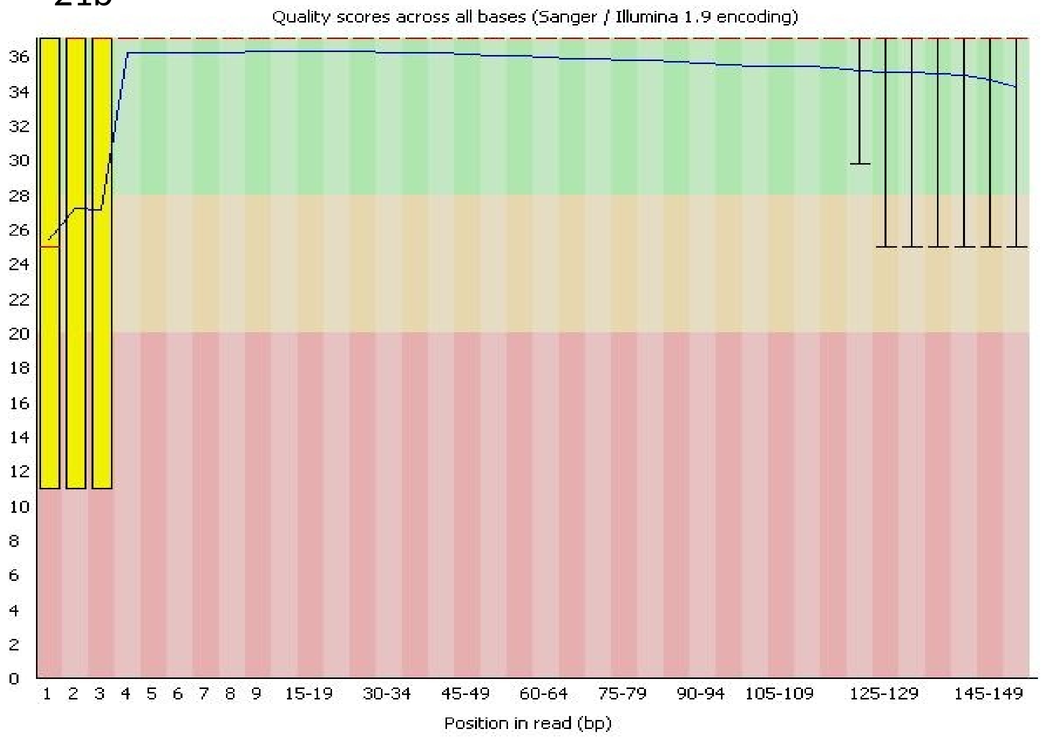

GDX_Male_5_R2

<u>Supplementary Figures 21a and 21b GDX Male #5</u>. FASTQ quality assessment.

Box and whisker plots illustrate the distribution of per base quality for left (R1; 21a) and right (R2; 21b) position reads. The blue line indicates mean value and the yellow box represents interquartile range (25%-75%). Upper and lower whiskers indicate 10% and 90% points respectively. Plots were generated by FASTQC program.

22a

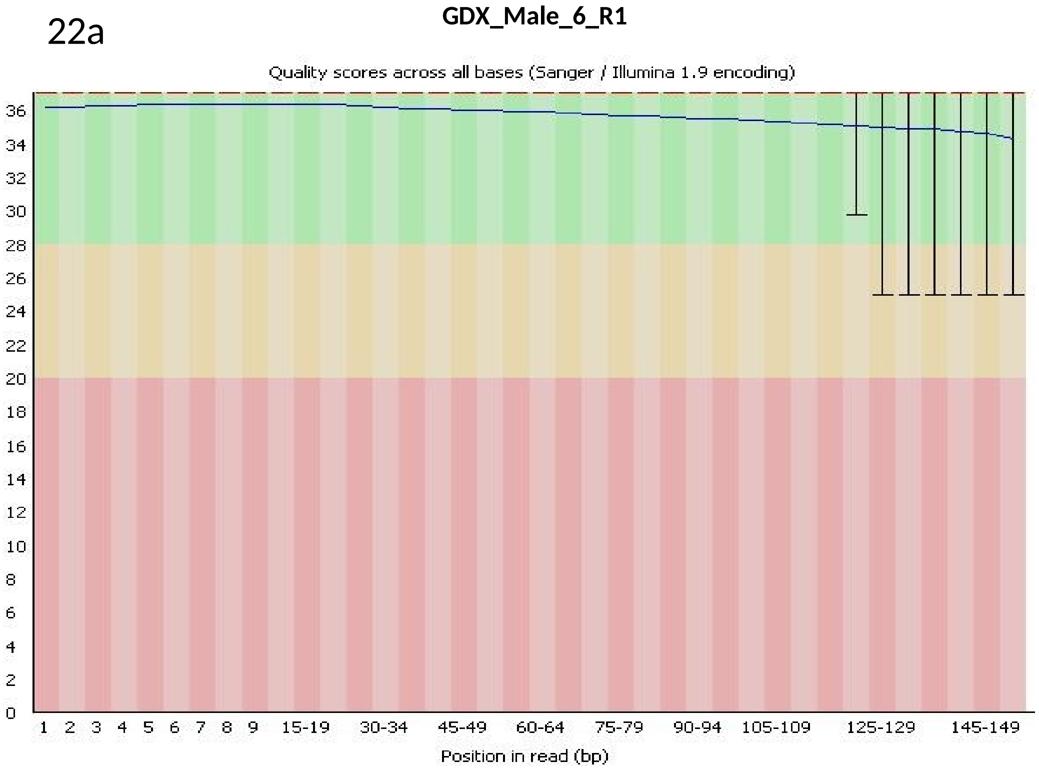

22b

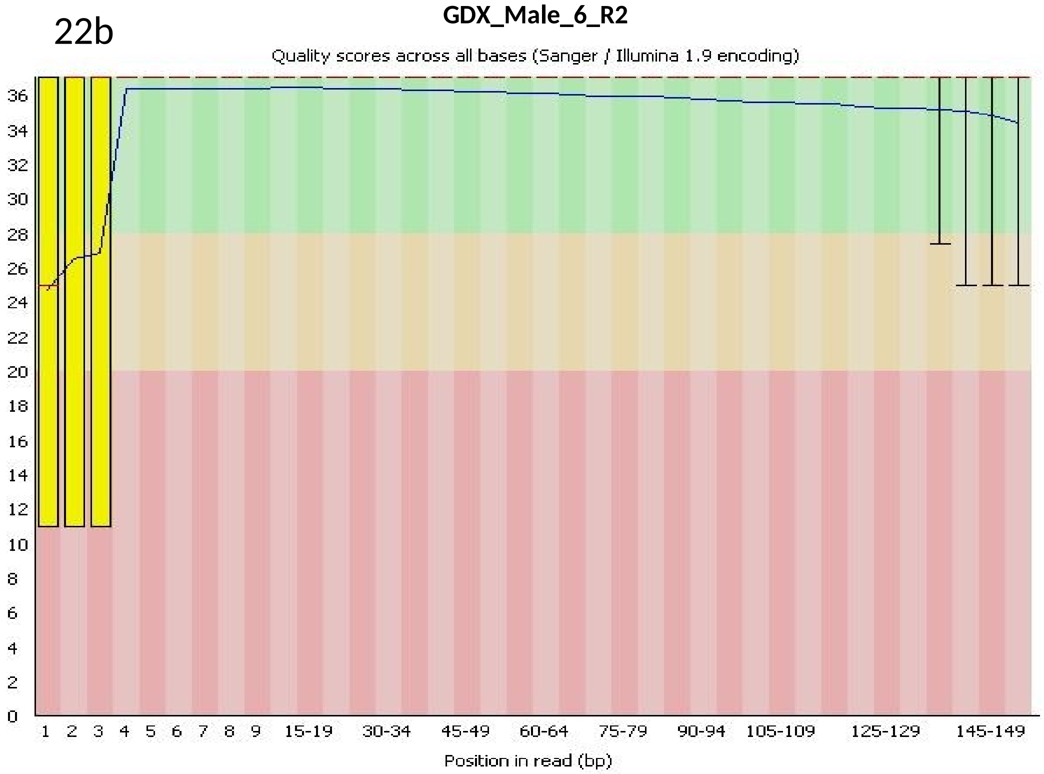

GDX_Male_6 GDX_Male_6_R2

<u>Supplementary Figures 22a and 22b GDX Male #6</u>. FASTQ quality assessment.

Box and whisker plots illustrate the distribution of per base quality for left (R1; 22a) and right (R2; 22b) position reads. The blue line indicates mean value and the yellow box represents interquartile range (25%-75%). Upper and lower whiskers indicate 10% and 90% points respectively. Plots were generated by FASTQC program.
